# Novel Exosome Biomarker Candidates for Alzheimer’s Disease Unravelled Through Mass Spectrometry Analysis

**DOI:** 10.1007/s12035-022-02762-1

**Published:** 2022-02-25

**Authors:** Tânia Soares Martins, Rui Marçalo, Cristóvão B. da Cruz e Silva, Dário Trindade, José Catita, Francisco Amado, Tânia Melo, Ilka Martins Rosa, Jonathan Vogelgsang, Jens Wiltfang, Odete A. B. da Cruz e Silva, Ana Gabriela Henriques

**Affiliations:** 1 Neuroscience and Signalling Group, Department of Medical Sciences, Institute of Biomedicine (iBiMED), University of Aveiro (UA), 3810-193 Aveiro, Portugal; 2 Laboratory of Instrumentation and Experimental Particle Physics-LIP, Av. Elias Garcia 14-1º, 1000-149 Lisbon, Portugal; 3 CEBIMED—Faculty of Health Sciences, University Fernando Pessoa, 4249-004 Porto, Portugal; 4 Paralab SA, 4420-437 Gondomar, Portugal; 5 Department of Chemistry, QOPNA (Organic Chemistry Natural and Agrofood Products and LAVQ REQUIMTE), University of Aveiro, 3810-193 Aveiro, Portugal; 6 Department of Psychiatry and Psychotherapy, University Medical Center Goettingen (UMG), Georg-August University, Von-Siebold-Str. 5, 37075 Goettingen, Germany; 7 Translational Neuroscience Laboratory, McLean Hospital, Harvard Medical School, Belmont, MA 02478 USA; 8 German Center for Neurodegenerative Diseases (DZNE), Von-Siebold-Str. 3a, 37075 Goettingen, Germany

**Keywords:** Alzheimer’s disease, Biomarkers, Blood, Diagnosis, Exosomes, Extracellular vesicles

## Abstract

Exosomes are small extracellular vesicles (EVs) present in human biofluids that can transport specific disease-associated molecules. Consequently blood-derived exosomes have emerged as important peripheral biomarker sources for a wide range of diseases, among them Alzheimer’s disease (AD). Although there is no effective cure for AD, an accurate diagnosis, relying on easily accessible peripheral biofluids, is still necessary to discriminate this disease from other dementias, test potential therapies and even monitor rate of disease progression. The ultimate goal is to produce a cost-effective and widely available alternative, which can also be employed as a first clinical screen. In this study, EVs with exosome-like characteristics were isolated from serum of Controls and AD cases through precipitation- and column-based methods, followed by mass spectrometry analysis. The resulting proteomes were characterized by Gene Ontology (GO) and multivariate analyses. Although GO terms were similar for exosomes’ proteomes of Controls and ADs, using both methodologies, a clear segregation of disease cases was obtained when using the precipitation-based method. Nine significantly different abundant proteins were identified between Controls and AD cases, representing putative biomarker candidate targets. Among them are AACT and C4BPα, two Aβ-binding proteins, whose exosome levels were further validated in individuals from independent cohorts using antibody-based approaches. The findings discussed represent an important contribution to the identification of novel exosomal biomarker candidates useful as potential blood-based tools for AD diagnosis.

**Supplementary Information:**

The online version contains supplementary material available at 10.1007/s12035-022-02762-1.

## Introduction

Alzheimer’s disease (AD) is the most common form of dementia worldwide, and the number of individuals affected by this condition is expected to increase exponentially in the next decades. At the histopathological level, AD is characterized by the aggregation and accumulation of the amyloid-beta (Aβ) peptide into senile plaques and of the microtubule-associated protein Tau into neurofibrillary tangles [1]. These deposits occur as a consequence of abnormal phosphorylation events [2–4], and lead to neuronal death and synaptic dysfunction, as well as glial activation and neuroinflammation [5], among other neurodegenerative events.

Despite the huge research efforts in the field, no effective treatment or cure is available thus far, and diagnosis is also challenging. AD diagnosis is based on clinical symptom evaluation, cognitive testing and brain imaging and, in some cases, supported by the molecular diagnostics, namely the monitoring of the gold standard biomarker triplet (Aβ, total-Tau and P-Tau 181) in the cerebrospinal fluid (CSF) [6, 7]. The latter neurochemical analysis has undeniable value in assisting AD differential diagnosis; nonetheless, it requires a lumbar puncture which is an invasive procedure, thus limiting its wide routine use or resource as a first screening tool. Hence, extensive research centered on the identification of biomarkers resorting to more easily accessible biofluids, like blood. Indeed, different molecular contents, including the biomarker triplet, have been tested in this peripheral biofluid [8]. Although currently, there are no reliable blood-based biomarkers for AD, this is urgently needed in clinical practice.

Recently, focus has been given to blood-derived exosomes in AD, a subclass of extracellular vesicles (EVs) with a multivesicular endosomal origin, ranging from 30 to 150 nm in diameter. These nanovesicles are formed by the inward budding of the endosome membrane and further released through the fusion of multivesicular bodies with the plasma membrane [9]. Exosomes are secreted by various cell types and can carry relevant proteins, lipids and nucleic acids, reflecting the status of the cells from which these nanovesicles derived. These type of EVs, present in various body fluids besides blood (e.g. saliva or urine), would represent easily accessible and cost-effective tools as biomarker sources for monitoring disease status, but also as drug delivery vehicles for a set of diseases, including cancer and inflammatory and neurological diseases [10]. The lipid bilayer of exosomes ensures the stability of the cargo, protecting the content from enzyme degradation in the bloodstream. In addition, since exosomes are capable of crossing the blood–brain barrier due to their small size, these nanovesicles can be particularly useful to study brain-related disorders [11].

In an AD context, exosomes carry disease specific-related signatures and contribute to the spreading of the amyloidogenic peptide species [12]. The biomarker value of these blood-derived exosomal vesicles has been tested, focusing on the levels of Aβ and other amyloid precursor protein fragments, Tau and phosphorylated Tau species [13–15]. Synaptic proteins, inflammatory mediators, growth factors and lysosomal proteins also presented distinct expression patterns in exosomes from AD cases when compared to Controls [16–19]. In addition, specific microRNA [12, 20, 21], metabolic [22], or lipid profile [23] alterations have been reported.

In this work, mass spectrometry (MS) was used to identify new blood-derived exosomal biomarker candidates associated with AD-exosomal proteomes. MS is highly sensitive and, thus, allows an unbiased biomarker identification in biofluids [24]. Proteome profiles were characterized through Gene Ontology (GO) analysis, and putative exosomal biomarker candidates were identified and further validated for two distinct patient cohorts. Biomarker candidates that arise from this analysis can constitute novel tools, potentially useful in AD and/or dementia peripheral biofluids-based diagnostics.

## Materials and Methods

### Study Cohorts

Blood samples were collected from individuals enrolled in a primary care-based cohort (pcb-cohort), which comprises volunteer individuals from the Baixo Vouga region of Aveiro. The inclusion and exclusion criteria were defined, and volunteers were submitted to a battery of cognitive tests as previously described [25, 26]. The cognitive and functional performance of volunteers was categorized based on the score obtained in 2 cognitive tests, the Mini-Mental State Examination (MMSE) and the Clinical Dementia Rating (CDR). MMSE scale cutoffs were set according to Portuguese population: 0–2 years of literacy, cutoff = 22; 3–6 years of literacy, cutoff = 24; and ≥ 7 years of literacy, cutoff = 27. Scores below the cutoff indicate possible cognitive impairment (MMSE +), and scores equal or above were classified as normal (MMSE −). CDR scale applied scores between 0 and 3, where 0 accounts for normal, 1 for mild dementia, 2 for moderate and 3 for severe dementia stages.

The pcb-cohort, herein designated as the UA-cohort, included a subgroup of 32 individuals that scored CDR ≥ 1 and MMSE + (mean age 77.38 ± 9.17) and 9 clinically reported AD cases (1 AD only scored CDR = 1), (mean age 78.67 ± 5.07). Sex- and age-matched Controls (CDR = 0 and MMSE −) were randomly selected from the same cohort (*n* = 32, mean age 76.69 ± 8.07 and *n* = 9, mean age 77.56 ± 4.83).

Another independent cohort, established at the Department of Psychiatry and Psychotherapy at the University Medical Center Goettingen (UMG-cohort), was also used for biomarker candidate’s validation. The UMG-cohort study group comprises 12 age-matched Controls (mean age 67.58 ± 7.74) and 12 demented individuals clinically diagnosed as ADs (mean age 73.17 ± 10.66) according to the 2011 McKhann criteria, as previously described [27, 28]. The UMG-cohort is characterized by neuropsychological testing (Consortium to Establish a Registry for Alzheimer’s Disease—CERAD test battery), and AD diagnosis of these patients is supported by CSF biomarkers (CSF-NDD) and/or PET analysis (amyloid PET and/or FDG-PET). The CSF molecular biomarkers (Total-Tau, Phospho-Tau 181, Aβ42 and Aβ42/Aβ40 ratio) were monitored and cerebral imaging tests were also carried out.

### EV Isolation and Characterization

EVs, with exosome-like characteristics, were isolated from serum samples, as previously described [22, 29, 30]. Two distinct exosome isolation methods were used: the precipitation-based ExoQuick Serum Exosome Precipitation Solution (System Biosciences) (ExoQ) and the column-based ExoSpin Blood Exosome Purification Kit (Cell Guidance Systems) (ExoS). In brief, serum samples were centrifuged to remove cell debris and then incubated with the respective isolation reagent, followed by a centrifugation step to pellet the nanovesicles. For ExoQ, two exosome isolations were performed: one where the EVs were eluted in PBS for transmission electron microscopy (TEM) and Nanoparticle Tracking Analysis (NTA) and the other where the EVs were resuspended in RIPA buffer with protease inhibitors for MS and western blot (WB) analysis. For ExoS, the pellet was resuspended in PBS, passed through a purification column and eluted with PBS. Part of the resulting EVs was used to perform TEM and NTA, while the remaining EV suspension was also mixed with RIPA buffer with protease inhibitors, allowing subsequent analysis. All exosome-enriched suspensions were aliquoted and stored at − 20 °C prior to analyses. Controls and AD samples were subjected to the same procedure for each EV isolation method.

The exosome’s concentration and size distribution curves were assessed by NTA, using NanoSight NS300™ instrument and NTA 3.2 software (Malvern Instruments, UK), as previously described [29]. NTA analysis was carried out in duplicate for each sample, and four video recordings were acquired for each exosome preparation. The particle concentration was corrected by the dilution factor (1:1000).

Exosome-enriched suspensions from both cohorts were randomly selected for TEM analysis. Paraformaldehyde (2%) was added to the exosome suspensions in PBS, and then exosomes were allowed to adsorb in 75 mesh Formvar/carbon grids. A 3% phosphotungstic acid solution was added to perform the negative staining. TEM images were obtained using a Hitachi H-9000 transmission electron microscope at 300 kV and images were captured using a slow-scan CCD camera.

The protein concentration of exosomal preparations was determined by BCA protein assay, and 50 μg of total protein was loaded from each sample, for sodium dodecyl sulfate polyacrylamide gel electrophoresis (SDS-PAGE). Following gel transfer into a nitrocellulose membrane, immunodetection was carried out for the exosomal markers Hsp70, CD63 and RAB11 and for the negative exosomal marker calnexin. In brief, membranes were blocked in 5% non-fat dry milk and incubated with the primary antibodies: anti-HSP70 (1:500) (SPA-812), anti-CD63 (1:500) (sc-5275), anti-RAB11 (1:500) (610657; BD Transduction Laboratories) and anti-Calnexin (1:200) (ADI-SPA-860-J). The secondary antibodies used were the anti-mouse (7076S) or anti-rabbit IgG, HRP-linked antibody (7074S) (Cell Signaling Technology), and protein bands were detected using the chemiluminescence reagent ECL Select (GE Healthcare Life Sciences™). Images were acquired with the ChemiDoc™ gel imaging system (Bio-Rad).

### EV Mass Spectrometry Analysis

For MS analysis, EVs with exosome-like characteristics were isolated using ExoQ and ExoS. For each method, serum-derived exosomes were isolated from 5 sex- and age-matched Controls (mean age 77.4 ± 5.41) and 5 clinically diagnosed AD patients (mean age 77.8 ± 5.59) from the UA-cohort. Subsequent biomarker validation was carried out in a higher number of samples from the UA-cohort and the UMG-cohort.

For MS analyses, EV preparations in RIPA buffer (ExoQ) or PBS plus RIPA buffer (ExoS) were sonicated and protein was quantified through BCA assay, using the Pierce™ BCA Protein Assay Kit. Loading buffer (4 ×) containing β-mercaptoethanol was added to exosomal samples, normalized for protein content (25 µg per sample) and separated in a 5–20% gradient SDS-PAGE. The resulting gels were stained with Coomassie Blue, and each individual gel lane was excised and divided into smaller fragments, to facilitate sample digestion. The fragment corresponding to the albumin molecular weight (around 66 kDa) was excluded and thus not analysed by mass spectrometry. The purpose was to reduce biological sample complexity, containing high levels of albumin, which may interfere with detection of other proteins by MS. Gel fragments were washed with ammonium bicarbonate and acetonitrile, and the proteins were reduced with 10 mM DTT (45 min at 56 ºC) and alkylated with 55 mM iodoacetamide (30 min at RT). Then, gel pieces were washed again, allowed to dry and rehydrated in digestion buffer containing 12.5 µg/mL of sequencing grade–modified trypsin in ammonium bicarbonate. Tryptic digestion was performed as previously described [31], with minor modifications. Trypsin was added at an enzyme-to-substrate ratio of 1:30 (w/w) followed by an overnight incubation with 50 mM ammonium bicarbonate at 37 °C. The peptides were extracted by the addition of 5% formic acid (FA, Fluka) and 5% FA/50% ACN (20 min each wash, 2°C) and lyophilized in SpeedVac (Thermo Savant), and peptides were reconstituted in 40 μL 1% FA solution.

Samples were analysed with a QExactive Orbitrap Mass Spectrometer (Thermo Fisher Scientific, Bremen) through the EASY-Spray nano ESI source (Thermo Fisher Scientific, Bremen) that was coupled to an Ultimate 3000 (Dionex, Sunnyvale, CA) HPLC (high-pressure liquid chromatography) system. The trap (5 mm × 300 µm i.d.) and the EASY-spray analytical (150 mm × 75 µm) columns used were C18 PepMap100 (Dionex, LC Packings) having a particle size of 3 µm. Peptides were trapped at 30 μL/min in 96% solvent A (0.1% FA). Elution was achieved with the solvent B (0.1% formic acid/80% acetonitrile v/v) at 300 nL/min. The 92-min gradient used was as follows: 0–3 min, 96% solvent A; 3–70 min, 4–25% solvent B; 70–90 min, 25–40% solvent B; 90–92 min, 90% solvent B; 90–100 min, 90% solvent B; 101–120 min, 96% solvent A. The mass spectrometer was operated at 1.8 kV in the data-dependent acquisition mode. A MS2 method was used with a FT survey scan from 400 to 1600 m/z (resolution 70,000; AGC target 1E6). The 10 most intense peaks were subjected to HCD fragmentation (resolution 17,500; AGC target 5E4, NCE 28%, max. injection time 100 ms, dynamic exclusion 35 s).

### MS Data Analyses

Spectra were processed and analysed using Proteome Discoverer (version 2.2, Thermo), with the MS Amanda (version 2.0, University of Applied Sciences Upper Austria, Research Institute of Molecular Pathology) and Sequest HT search engines. The UniProt (TrEMBL and Swiss-Prot) protein sequence database (version of October 2017) was used for all searches under *Homo sapiens*. The database search parameters were as follows: carbamidomethylation of cysteine, oxidation of methionine and the allowance for up to two missed tryptic cleavages. The peptide mass tolerance was 10 ppm, and fragment ion mass tolerance was 0.02 Da. To achieve a 1% false discovery rate, the Percolator (version 2.0, Thermo) node was implemented for a decoy database search strategy and peptides were filtered for high confidence and a minimum length of 6 amino acids, and proteins were filtered for a minimum number of peptide sequences of 1. The obtained results were further filtered, applying a cutoff at 1.5-fold increase and another at 0.5-fold decrease. Also, abundances found in less than 2 out of 5 samples were not regarded as being present in the respective condition (AD or Control) and, when no abundance was measured for one of the groups of samples, a 100-fold increase/0.01-fold decrease was considered for the ratio.

### Bioinformatic Analysis of EVs

Proteomes obtained by MS (list of gene names from proteins identified) were initially overlapped through Venn diagrams with a serum exosomal gene list (Exo Serum list), obtained from databases and from literature search as described in [30], using the bioinformatics and evolutionary genomics website (http://bioinformatics.psb.ugent.be/webtools/Venn/; accessed on February 3, 2020), in order to determine the percentage of exosomal proteins present in EV samples analysed. Only MS proteins for which gene name was available were included in the overlap. From the exosomal proteomes obtained by MS, seven immunoglobulin chains were not included in this analysis since no gene name was available. Additionally, the set of proteins identified for Controls and ADs for each kit by MS (ExoQ and ExoS) was categorized according to their Gene Ontology (GO) annotation, obtained from the UniProt-Swiss-Prot database. The GO terms were filtered according to the Generic GO Slim, and categorization was carried out for ‘Molecular Function’ and ‘Biological Process’.

The protein lists identified by MS were then analysed through the use of a dedicated software framework (SysBioTK) as previously reported [2], with the exception of the partial least square (PLS) analysis.

Data was prepared independently for each analysis (AD vs Control for ExoQ; AD vs Control for ExoS). In a first step, the protein abundances obtained from MS were normalized by the median of the protein abundances of the sample. Subsequently, the abundances were independently transformed for each protein in each sample through the use of the binary logarithm. For some proteins, in some samples, there was no abundance data from MS; regardless, these proteins were not removed. The prepared data was then converted into tabular format and exported into a text file, for later use with MetaboAnalyst 4.0 [32] for the PLS analysis (performed on February 11, 2020), in order to maintain a consistent data set. PLS analysis was performed to evaluate which kit had the highest discriminatory capacity between Controls and AD cases.

To identify proteins with statistically significant differences in abundance, a Welch’s *t*-test with a significance level of 5% (*α* = 0.05) was applied to the mean ‘normalized and transformed abundance’ of the protein in each condition (i.e. the mean across samples for each condition). Volcano plots were created by plotting, for each protein, the p-value of the Welch’s *t*-test against the fold increase of the mean ‘normalized abundance’ of the protein, calculated as the base 2 logarithm of the ratio of the mean normalized abundance in AD conditions to the mean normalized abundance in Control conditions. The fold change threshold was set to 2, and a line representing the 5% significance level was drawn.

Heatmaps were created by taking the ‘normalized abundance’ for each protein in each sample. The dendrograms were calculated using the Ward method to cluster similar samples and proteins together. A Euclidian distance metric was used to calculate the distances for the Ward method. The color scale represents the ‘normalized abundance’.

### EV Biomarker Candidate Analyses

Following MS and bioinformatic analysis, biomarker validation was then carried out in a higher number of samples from the UA- and the UMG-cohorts. WB analyses were performed to assess the patterns of two biomarker candidates identified by MS: alpha-1-antichymotrypsin (AACT) and C4b-binding protein alpha chain (C4BPα). The protein concentration of exosome samples isolated with ExoQ were determined, and 50 µg of protein was loaded, per sample, in a 5–20% SDS-PAGE followed by protein transfer to nitrocellulose membranes. Membranes were then blocked with 5% non-fat dry milk and incubated with the primary antibodies anti-AACT (1:500) (sc-59430; Santa Cruz Biotechnology) and anti-C4BPα Antibody (1:500) (sc-398720; Santa Cruz Biotechnology). Subsequently, the membranes were incubated with the anti-mouse IgG, HRP-linked antibody (1:2000) (Cell Signaling Technology). Protein bands were detected, as described above, using the chemiluminescence reagent ECL Select (GE Healthcare Life Sciences™), images were acquired with the ChemiDoc™ gel imaging system (Bio-Rad), and protein bands were quantified using the Image Lab software (v 6.0.1, Bio-Rad). AACT or C4BPα band densitometry values for each individual sample were normalized to an exosomal pool loaded in every membrane. Graphs express the relative density ratios. Further, AACT and C4BPα levels were also evaluated by enzyme-linked immunosorbent assay (ELISA), in serum-derived exosomes of the same individuals, using the commercial Human AACT ELISA Kit (ab217779; Abcam) or the Human C4 binding protein A ELISA Kit (NBP2-60,550; Novus Biologicals), according to manufacturer’s instructions. EV samples were diluted, and equal amounts of protein were used for AACT or C4BPα quantification.

### Statistical Analysis

Statistical analysis was carried out using SPSS version 27 (IBM) or GraphPad Prism 7 (GraphPad Software, La Jolla, CA, USA). Data distribution was assessed by the Shapiro–Wilk test, and homogeneity of variance was assessed using Levene’s test. Kolmogorov–Smirnov was used to compare the particle size distribution. Exosome concentrations were compared using the non-parametric Kruskal–Wallis, and mode sizes were compared by means of a one-way ANOVA with the Bonferroni post hoc test. Levels of biomarker candidates were compared using unpaired *t*-tests. *p*-values ≤ 0.05 were considered significant.

## Results

### EV Isolation from Controls and AD Cases

**Fig. 1:**
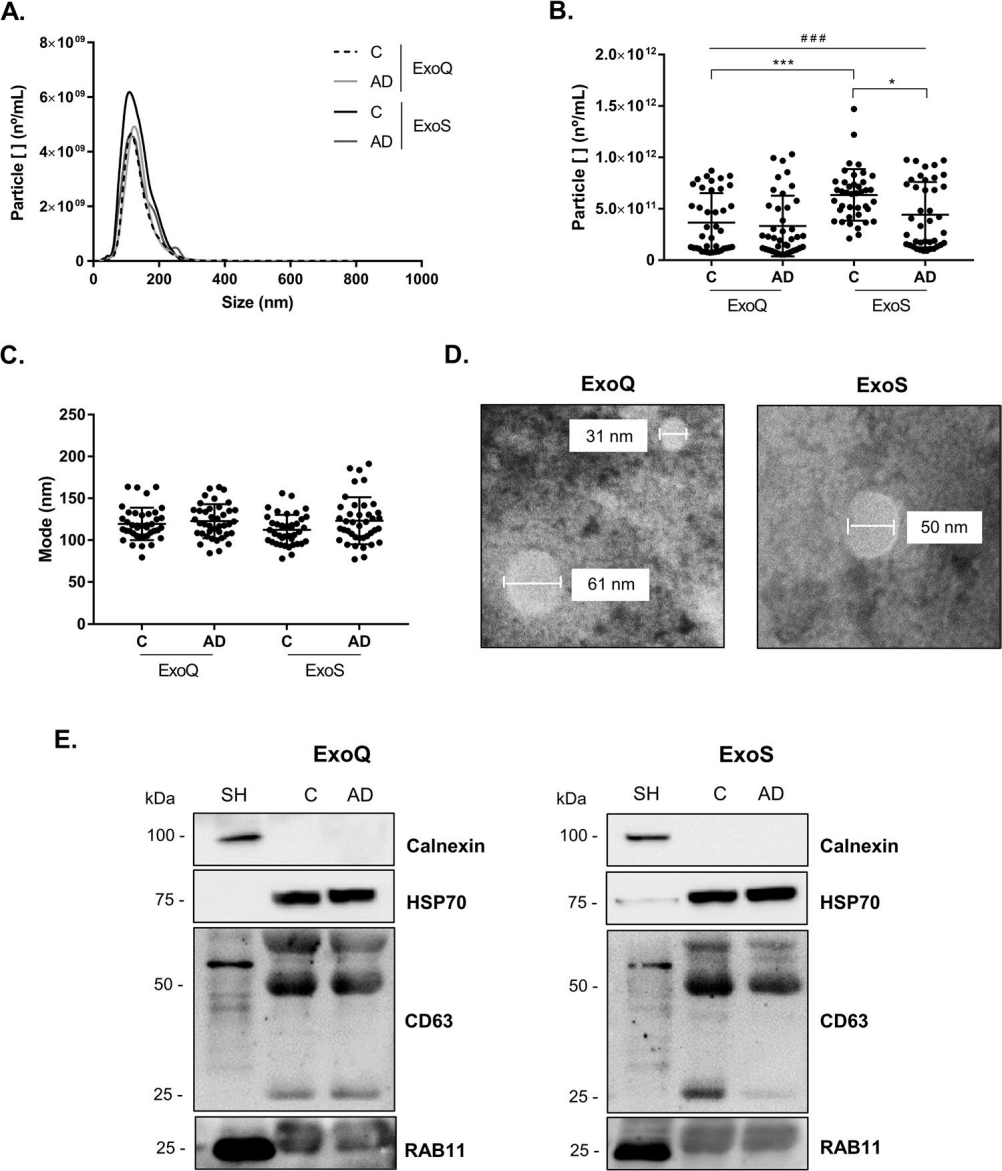
Characterization of blood-derived EVs isolated using ExoQ and ExoS. Exosome-like EVs size distribution (**a**), particle concentration (**b**) and mode size (**c**) determined by Nanoparticle Tracking Analysis. **d** Transmission electron microscopy of isolated nanovesicles and **e** western blot analysis of the exosomal markers HSP70, CD63 and RAB11. EVs were isolated from UA-Cohort serum samples. Particle concentrations were compared using non-parametric Kruskal–Wallis and mode sizes using one-way ANOVA with Bonferroni post hoc test. Abbreviations: ExoQ, ExoQuick; ExoS, ExoSpin; SH, SH-SY5Y lysates. ^###^*p* ≤ 0.001, among the four groups; ***p ≤ 0.001 between Controls of ExoQ and ExoS; *p ≤ 0.05 between Controls and AD isolated using ExoS

Prior to MS analysis, EVs with exosome-like characteristics were isolated from Control and AD cases using two distinct approaches, the precipitation-based (ExoQ) and column-based (ExoS) isolation kits. Exosomes obtained were characterized by Nanoparticle Tracking Analysis (Fig. 1a–c), and size distribution curves revealed that both kits isolated vesicles within the expected size range for exosomes. For ExoQ, no differences were found in the particle concentration between Controls (3.64 × 10^11^ ± 2.69 × 10^11^ particles/mL) versus AD cases (3.33 × 10^11^ ± 2.81 × 10^11^ particles/mL) or in the mode size between the Control and AD group (119.5 ± 17.77 nm and 122.7 ± 18.40 nm, respectively) (Fig. 1a–c). In contrast, for ExoS the size distribution of the isolated vesicles and the particle concentration were significantly different (*p* ≤ 0.05) between the Control (6.36 × 10^11^ ± 2.49 × 10^11^ particles/mL) and AD (4.41 × 10^11^ ± 3.15 × 10^11^ particles/mL) groups, although no significant differences were found between the mode size of vesicles isolated from Control (112.5 ± 17.32 nm) and AD groups (123.3 ± 28.48 nm). Differences for particle concentrations were also found between the Controls of ExoQ and ExoS and among the four groups (*p* ≤ 0.001). Consistently with NTA, TEM analysis revealed that both methods isolated exosomes with the expected morphology and size (Fig. 1d).

To confirm the nature of the nanovesicles, WB analysis was performed using pooled samples from Controls and AD cases. The exosomal markers HSP70, CD63 and RAB11 were detected in the vesicles isolated using ExoQ and ExoS (Fig. 1e), while the negative exosome marker calnexin was not detected.

### GO Analyses of EVs from Controls and AD Cases

As explained above, serum-derived EVs were isolated from Controls and AD cases using both ExoQ and ExoS and then characterized by MS analysis. ExoQ renders a higher total number of proteins identified by MS when compared to ExoS (*p* ≤ 0.01). In exosome-like particles isolated with ExoQ, an average of 136 proteins were identified in the Control group and 117 proteins were identified in AD cases. For ExoS, an average of 100 proteins were found in Controls and 85 proteins were identified in ADs (Table 1; Supplementary Tables 1 and 2), including protein isoforms. For both kits, AD cases presented a small decrease in the number of proteins identified by MS, although not significantly different. The common proteins found in exosomes isolated from Controls and ADs using both ExoQ and ExoS are presented in Supplementary Table 3.

**Table 1:** Number of exosomal proteins identified by mass spectrometry for both kits. Exosome-like particles were isolated with ExoQ or ExoS, followed by mass spectrometry analysis. Abbreviations: ExoQ, ExoQuick; ExoS, ExoSpin; SD, standard deviation

	ExoQ		ExoS	
	Controls	AD	Controls	AD
Mean ± SD	136.8 ± 7.5	117.8 ± 29.5	100.2 ± 8.0	85.8 ± 16.6

**Fig. 2:**
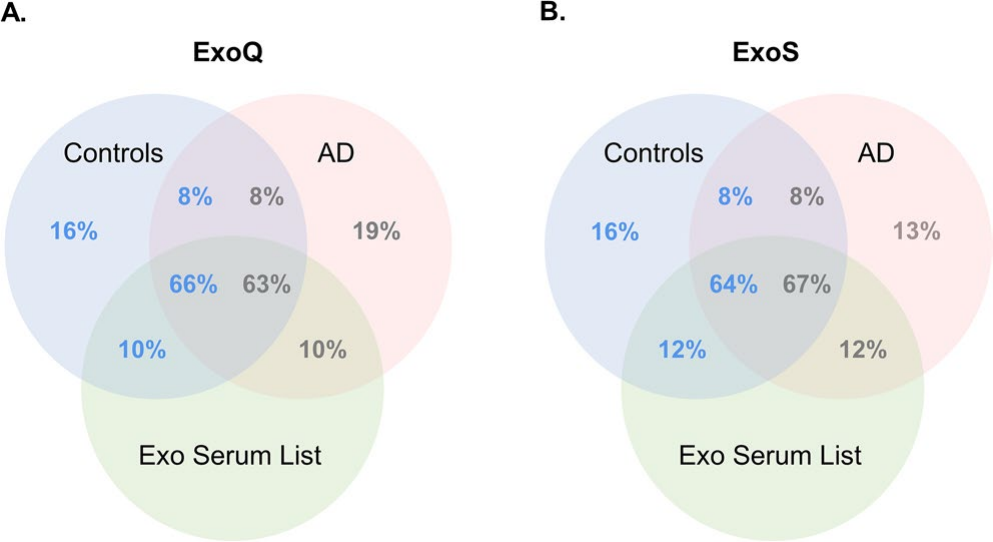
Overlap of exosomal-enriched proteomes obtained by MS with serum-derived exosomal database recovered list. Venn diagrams illustrating the overlap of the exosomal-enriched proteomes obtained by MS (list of gene names from proteins identified) after exosome isolation from serum samples of the UA-Cohort, using ExoQ (**a**) and ExoS (**b**), with the serum exosomal gene list (Exo Serum List) collated from databases and literature search. Abbreviations: ExoQ, ExoQuick; ExoS, ExoSpin

For further analyses, exosomal proteomes obtained through MS (list of gene names from proteins identified) were overlapped with an ‘in silico’ serum-derived exosomal gene list. This list was collated using public databases, namely Vesiclepedia, EVpedia and Exocarta and complemented following a literature review on exosomal AD-related proteins [12, 30]. More than 70% of the exosomal proteins identified by MS and isolated either with ExoQ or ExoS were also present in the serum-derived exosomal gene list, reinforcing the exosomal nature of the samples (Fig. 2; Supplementary Table 4 and 5).

**Fig. 3:**
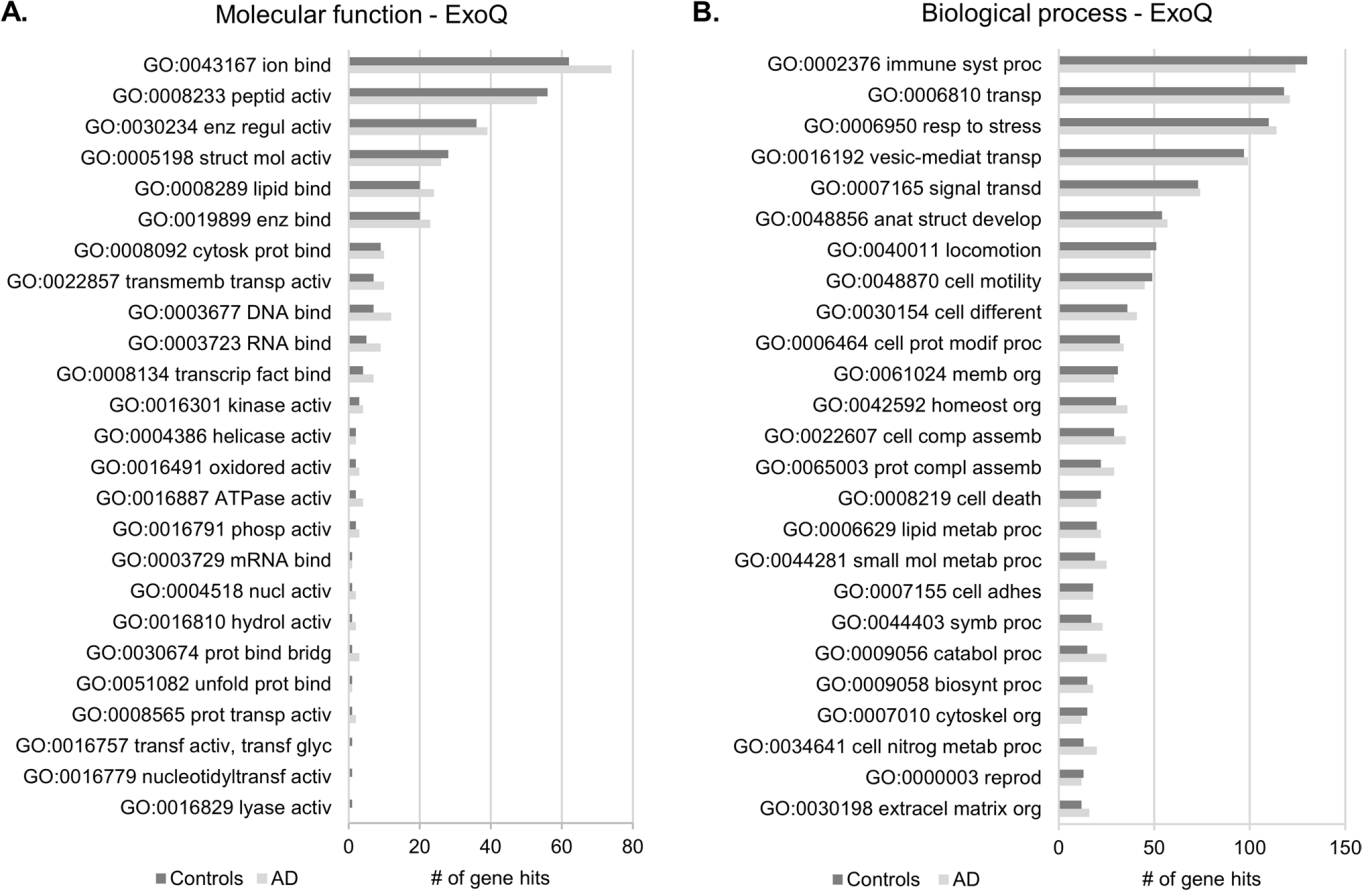

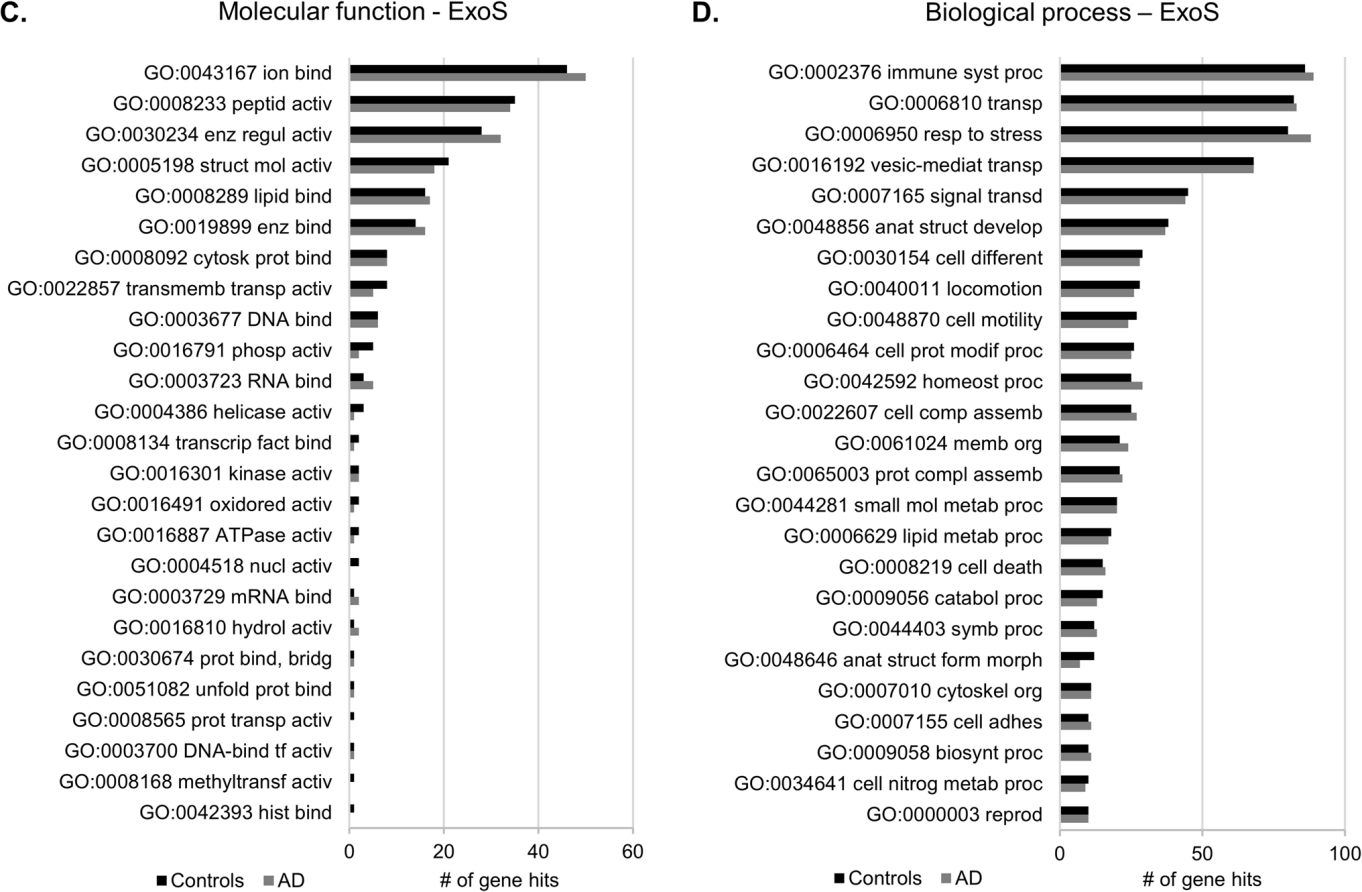
GO analysis of Controls and AD exosomal-enriched proteomes obtained with ExoQ or ExoS. The top 25 molecular function and biological processes terms were annotated for exosomes isolated from serum samples of the UA-Cohort, with ExoQuick (**a**, **b**) or ExoSpin (**c**, **d**). Dark gray bars represent Controls, and light gray bars represent AD cases. Abbreviations: ExoQ, ExoQuick; ExoS, ExoSpin. Abbreviations of GO molecular function: GO:0043167, ion binding; GO:0008233, peptidase activity; GO:0030234, enzyme regulator activity; GO:0005198, structural molecule activity; GO:0008289, lipid binding; GO:0019899, enzyme binding; GO:0008092, cytoskeletal protein binding; GO:0022857, transmembrane transporter activity; GO:0003677, DNA binding; GO: 0003723, RNA binding; GO:0008134, transcription factor binding; GO:0016301, kinase activity; GO:0004386, helicase activity; GO:0016491, oxidoreductase activity; GO:0016887, ATPase activity; GO:0016791, phosphatase activity; GO:0003729, mRNA binding; GO:0004518, nuclease activity; GO:0016810, hydrolase activity, acting on carbon–nitrogen (but not peptide) bonds; GO: 0030674, protein binding, bridging; GO:0051082, unfolded protein binding; GO:0008565, protein transporter activity; GO:0016757, transferase activity, transferring glycosyl groups; GO:0016779, nucleotidyltransferase activity; GO:0016829, lyase activity; GO:0003700, DNA-binding transcription factor activity; GO:0008168, methyltransferase activity; GO:0042393, histone binding. Abbreviations of GO biological process: GO:0002376, immune system process; GO:0006810, transport; GO:0006950, response to stress; GO:0016192, vesicle-mediated transport; GO:0007165, signal transduction; GO:0048856, anatomical structure development; GO:0040011, locomotion; GO:0048870, cell motility; GO:0030154, cell differentiation; GO:0006464, cellular protein modification process; GO:0061024, membrane organization; GO:0042592, homeostatic process; GO:0022607, cellular component assembly; GO:0065003, protein-containing complex assembly; GO:0008219, cell death; GO:0006629 lipid metabolic process; GO:0044281, small molecule metabolic process; GO:0007155, cell adhesion; GO:0044403, symbiont process; GO:0009056, catabolic process; GO:0009058, biosynthetic process; GO:0007010, cytoskeleton organization; GO:0034641, cellular nitrogen compound metabolic process; GO:0000003, reproduction; GO:0030198, extracellular matrix organization; GO:0048646, anatomical structure formation involved in morphogenesis

GO functional enrichment analysis was performed to characterize the exosomal-enriched proteome of Controls and AD cases, obtained with ExoQ (Fig. 3a, b) or ExoS (Fig. 3c, d). The GO terms for molecular function and biological process were similar between Controls and AD cases of proteomes obtained from both kits. The top 5 molecular function terms were ion binding, peptidase activity, enzyme regulator activity, structural molecule activity and lipid binding, whereas the top biological process terms in both cases were immune system process, transport, response to stress, vesicle-mediated transport and signal transduction. In general, ExoQ renders in a higher number of proteins isolated and consequently more hits for each category. Although not statistically significant, differences in the number of the genes or specific GO terms could be found associated with each kit. These GO proteome differences may be interesting when addressing a particular process and/or approach during biomedical research.

### Partial Least Square Analysis of EV Proteomes

**Fig. 4:**
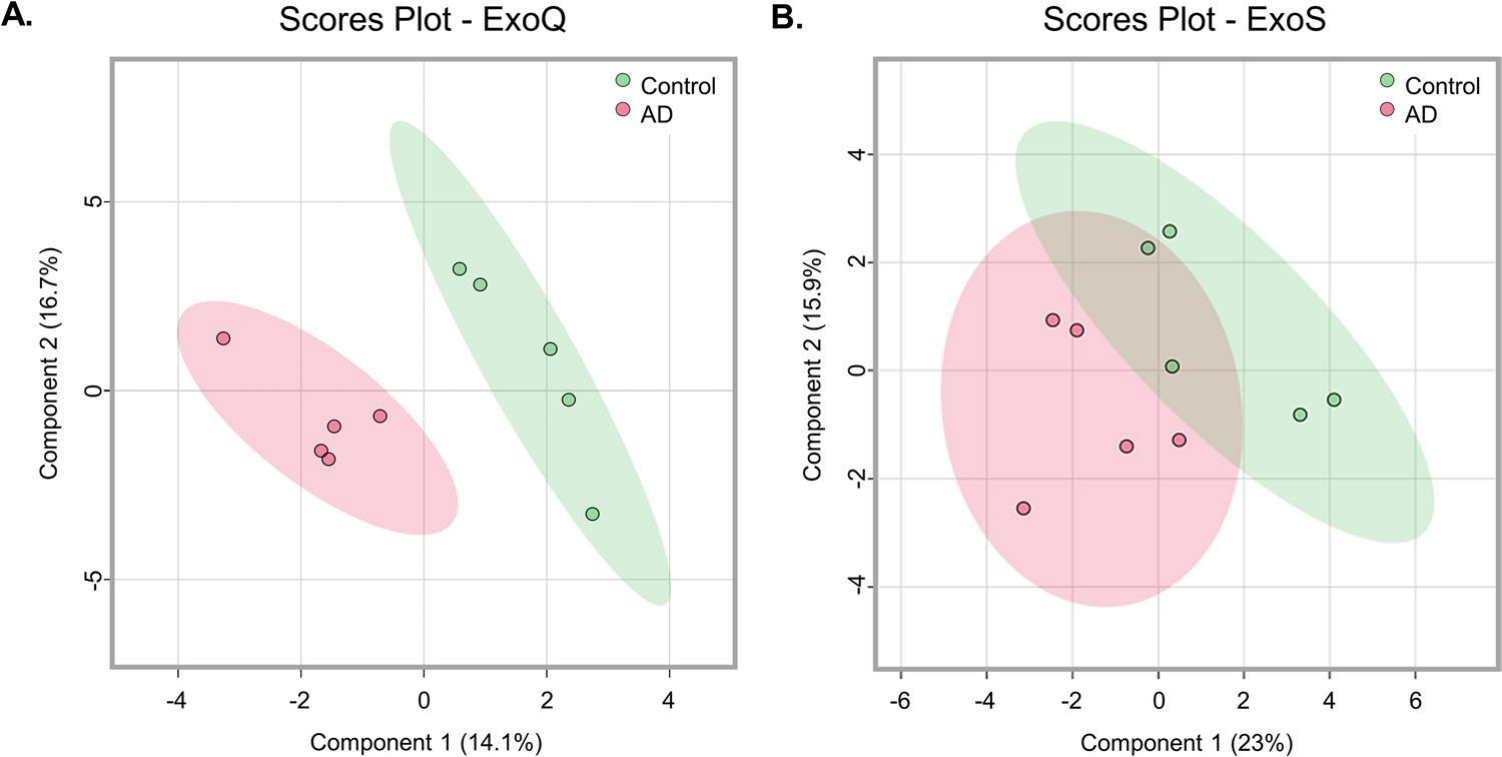
Partial least square analysis of EV proteomes of Controls and AD cases. EV preparations with exosome-like characteristics were isolated with ExoQ (**a**) or ExoS (**b**) from serum samples of the UA-Cohort. Green and pink areas represent the 95% confidence region. Abbreviations: ExoQ, ExoQuick; ExoS, ExoSpin

PLS analysis was also carried out to assess and compare the performance of the two kits in discriminating Control and AD cases (Fig. 4). This analysis revealed that ExoQ presents a higher discriminatory power, as there was no overlap between Controls and AD cases. Taking this evidence into consideration, and the fact that significant differences were obtained in the number of exosome particles isolated from Controls and AD cases, when using ExoS, subsequent analyses were performed focusing on the results obtained with ExoQ in both groups, AD and Controls. Nonetheless, data obtained for ExoS is also included as supplementary material.

### EV Proteomic Signature of Controls and AD Cases

**Fig. 5:**
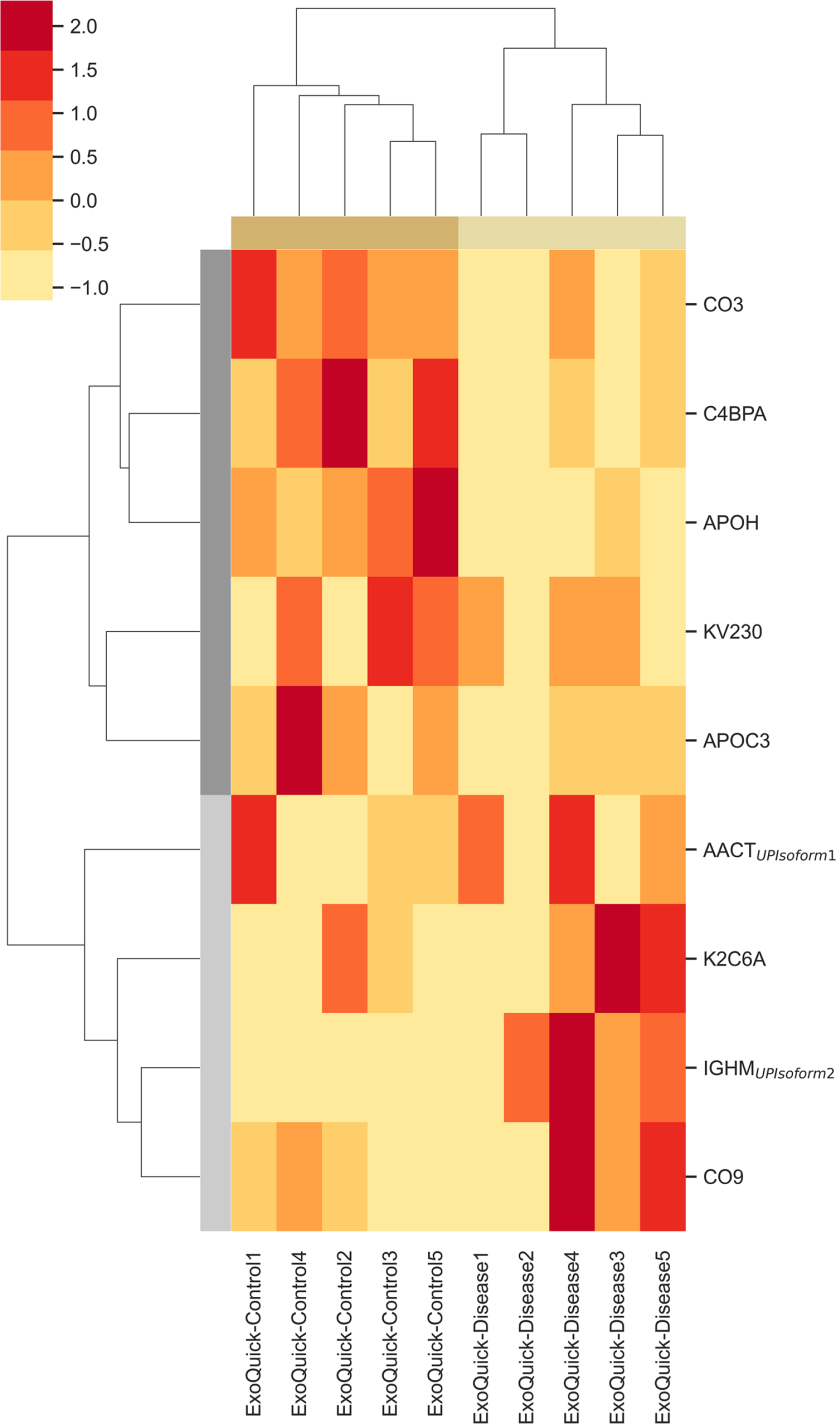
Heatmap of exosomal proteome abundance changes in disease. Heatmaps were constructed for the significantly different proteins identified in exosomes of Controls vs AD cases, isolated from serum samples of the UA-Cohort*.* Differences were determined using Welch’s *t*-test and a 95% confidence level was considered. The bars on the top of heatmaps show the kits category. Red represents higher abundance and light yellow represents lower abundance levels. Abbreviations: AACT, α-1-antichymotrypsin; APOC3, apolipoprotein C-III; APOH, beta-2-glycoprotein 1; C4BPA, C4b-binding protein alpha chain; CO3, complement C3; CO9, complement component 9; Disease, Alzheimer’s disease cases; IGHM, immunoglobulin heavy constant mu; K2C6A, keratin, type II cytoskeletal 6A; KV230, Immunoglobulin kappa variable 2–30

Heatmaps were constructed to assess which exosomal proteins could potentially be used to discriminate Controls from AD cases. This hierarchical analysis revealed 9 proteins with significantly different abundance levels between Controls and AD cases (Fig. 5).

**Fig. 6:**
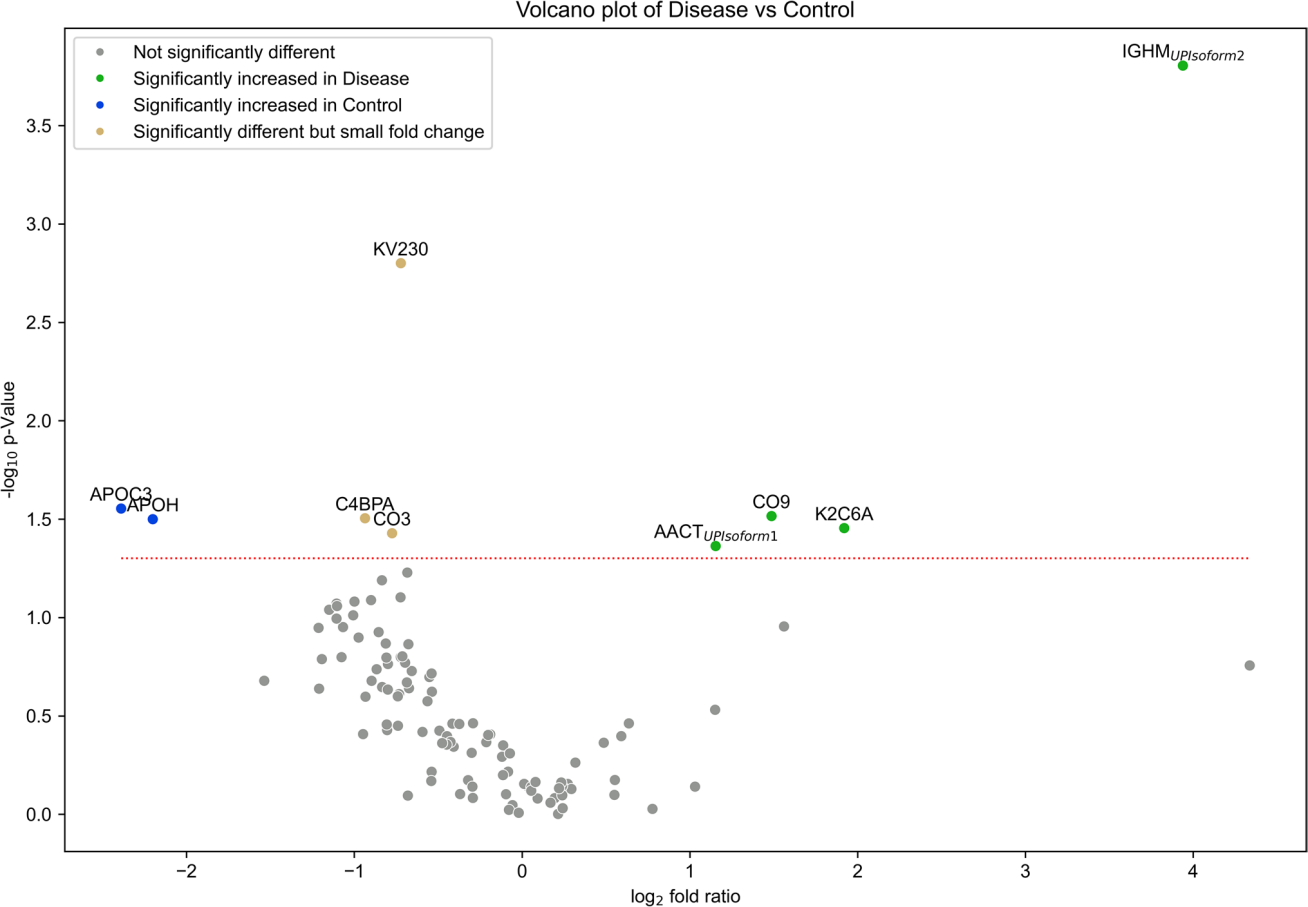
Volcano plot of significant different exosomal proteins in AD cases versus Controls. Exosomes were isolated from serum-samples of the UA-Cohort using ExoQuick. The dashed red line indicates the *p*-value threshold of 0.05. Abbreviations: AACT, α-1-antichymotrypsin; APOC3, apolipoprotein C-III; APOH, beta-2-glycoprotein 1; C4BPA, C4b-binding protein alpha chain; CO3, complement C3; CO9, complement component 9; Disease, Alzheimer’s disease cases; IGHM, immunoglobulin heavy constant mu; K2C6A, keratin, type II cytoskeletal 6A; KV230, immunoglobulin kappa variable 2–30

As depicted in the heatmap and in the volcano plot (Fig. 6), apolipoprotein C-III (APOC3), beta-2-glycoprotein 1 (APOH), C4b-binding protein alpha chain (C4BPα), Complement C3 (CO3) and immunoglobulin kappa variable 2–30 (KV230) were significantly increased in Control individuals, while alpha-1-antichymotrypsin (AACT) isoform 1, complement component 9 (CO9), immunoglobulin heavy constant mu (IGHM) Isoform 2 and keratin, type II cytoskeletal 6A (K2C6A) were significantly increased in AD cases. Indeed, 6 of these 9 proteins have already been described as altered in the context of AD (AACT, APOC3, APOH, C4BPα, CO3 and CO9) (Table 2).

**Table 2:** Significant different exosomal proteins and their role in AD. Exosomes were isolated with ExoQ prior to MS analysis

	UniProt ID	Gene	Protein name	Alteration in AD	Involvement in AD pathogenesis	Biofluid/brain	Reference
ExoQ	P01011-1 (AACT_HUMAN)	SERPINA3	α-1-Antichymotrypsin	↑	Present at amyloid plaques	Human brain	[38, 42, 43, 37]
				↑		CSF and serum	[44, 45]
				=		CSF and serum	[46–49]
				=		Serum	[50]
				↑		Serum	[51–54]
				↑		CSF and plasma	[39, 55]
				↑		Plasma	[56, 57]
					Promotes Aβ plaques deposition	Transgenic mouse brain	[40]
					Induce tau hyperphosphorylation	Transgenic mouse brain	[37, 41]
	P02656 (APOC3_HUMAN)	APOC3	Apolipoprotein C-III	↓		Plasma	[58]
	P02749 (APOH_HUMAN)	APOH	Beta-2-glycoprotein 1	↑		CSF	[59]
				↓ in ApoE4 carriers and MCI individuals		Plasma	[60]
	P04003 (C4BPA_HUMAN)	C4BPA	C4b-binding protein alpha chain*		Detected in Abeta plaques and apoptotic cells	Human brain	[33]
				=		CSF and plasma	
					Bind to Aβ through α chain; limits the complement activation on Aβ peptide	In vitro	
				↓		Plasma	[61]
	P01024 (CO3_HUMAN)	C3	Complement C3	↑		Human AD brain and CSF	[62]
					C3 deficiency protected against synapse loss and cognitive decline		[63]
				↓	Low levels of C3 associated with a higher risk of AD	Plasma	[64]
				C3b, C3d ↑		Plasma astrocyte-derived exosomes	[17, 18]
	P02748 (CO9_HUMAN)	C9	Complement component C9	↑	Increased number of C9-stained diffuse plaques in AD vs C or MCI	Postmortem human brain specimens	[65]
				↑		Human brain	[66]
				C5b-C9 ↑		Plasma astrocyte-derived exosomes	[17, 18]
	P01871-2 (IGHM_HUMAN)	IGHM	Immunoglobulin heavy constant mu	-	-	-	-
	P02538 (K2C6A_HUMAN)	KRT6A	Keratin, type II cytoskeletal 6A	-	-	-	-
	P06310 (KV230_HUMAN)	IGKV2-30	Immunoglobulin kappa variable 2–30	-	-	-	-

^*^The information found refers only to C4BP, and it is not specific to the alpha chain

Of the 9 candidates, two were selected for subsequent validation: AACT and C4BPα. These were found to be the most interesting candidates since both AACT and C4BPα are detected in senile plaques and both are Aβ-binding proteins [33–36]. AACT can also induce tau phosphorylation [37]. In addition, the patterns of these candidates have been previously addressed in different biofluids, namely CSF, plasma and/or serum. Although some inconsistencies have been observed (Table 2), previous studies reported similar patterns as those obtained here for serum-derived exosomes in MS analysis. Thus, these candidates with opposite expression patterns in Controls and AD cases were further validated by WB and ELISA in exosomes.

Heatmaps were also obtained for the exosomal proteome corresponding to the ExoS method from which 4 proteins showed significant differences for the abundance values among the Controls and AD cases (HV374, ITIH4 isoform 1, THRB and HRG) (Supplementary Fig. 1 and 2, respectively). However, since the disease discriminatory power of this kit was lower than for ExoQ, these candidates were not further pursued.

### Validation of the Putative EV Candidates

**Fig. 7:**
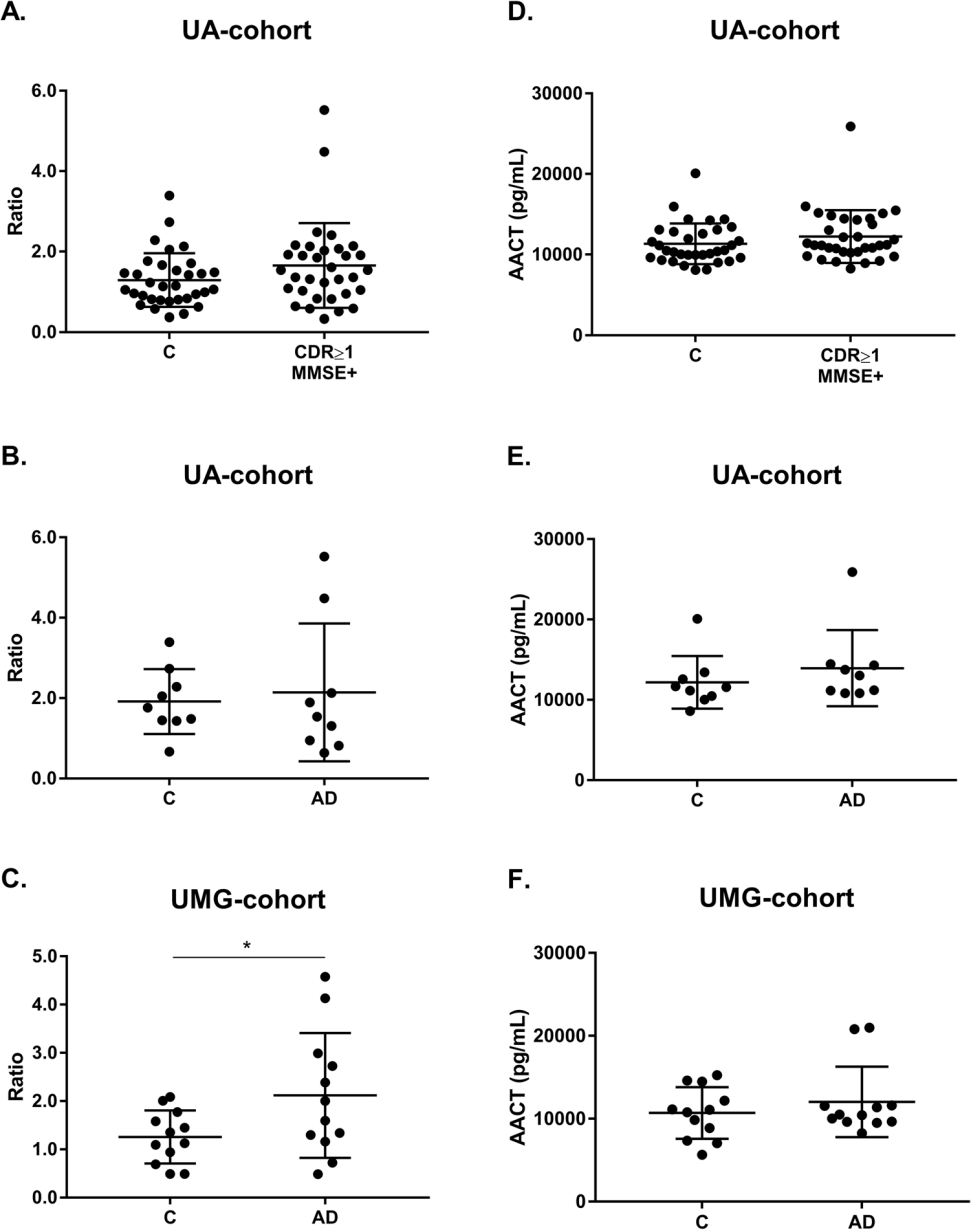
AACT exosomal levels in dementia and AD cases monitored by distinct antibody-based approaches. AACT levels were assessed through immunoblot analysis or commercial ELISA assays in serum-derived exosomes from Controls (CDR = 0 and MMSE −) and individuals with dementia (CDR ≥ 1 and MMSE +) from UA-cohort (**a**, **d**), and AD clinically diagnosed cases from UA-cohort (**b**, **e**) or UMG-cohort (**c**, **f**). For WB, each point represents the relative densitometry ratio. For ELISA, each point represents the mean concentration value obtained for each individual. The solid horizontal line shows mean, and error bars indicates standard deviations. Abbreviations: AD, Alzheimer’s disease; C, Controls; CDR, Clinical Dementia Rate; MMSE, Mini-Mental State Examination. *p ≤ 0.05

**Fig. 8:**
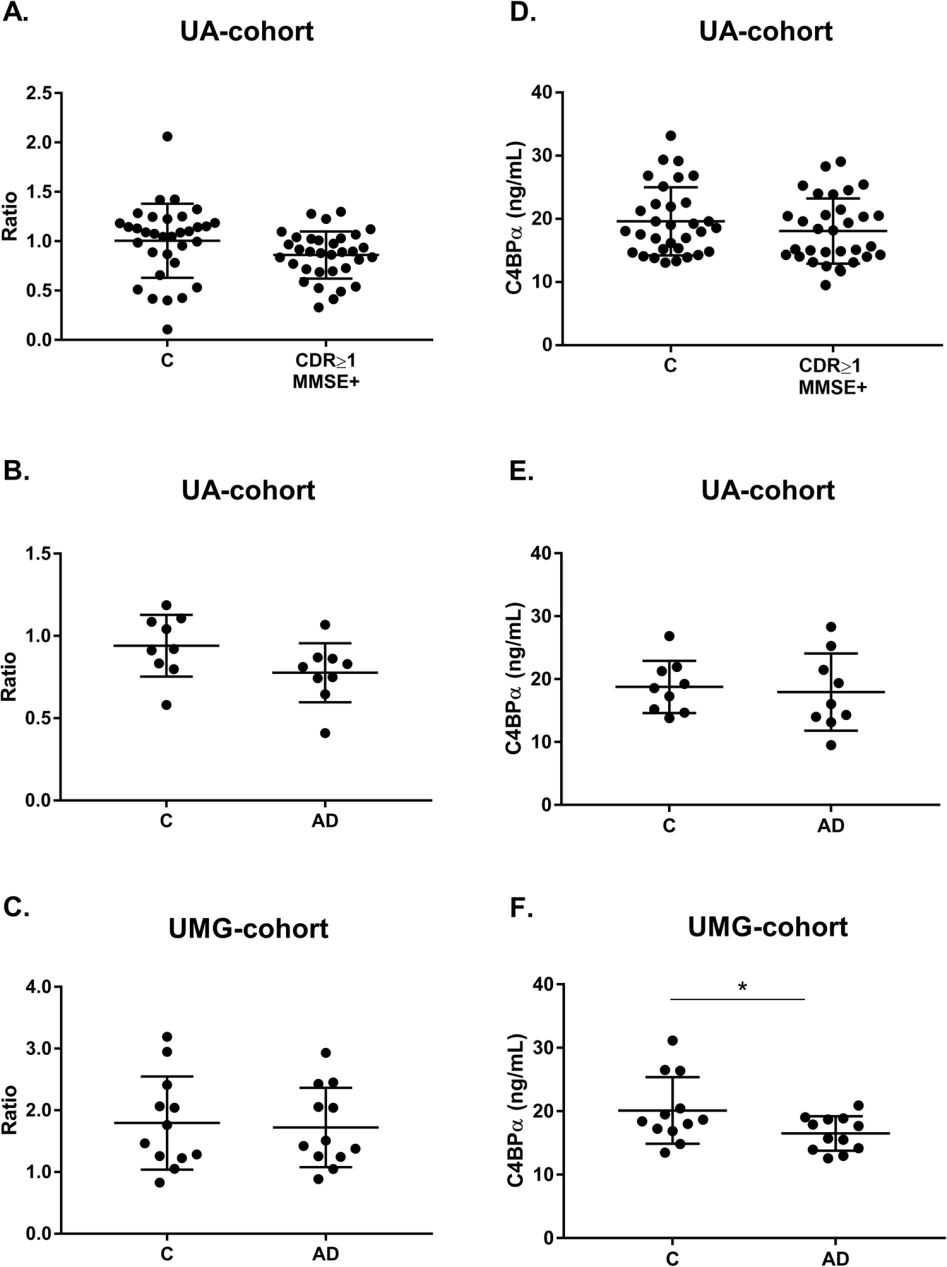
C4BPα exosomal levels in dementia and AD cases monitored by distinct antibody-based approaches. C4BPα levels were assessed through immunoblot analysis or commercial ELISA assays in serum-derived exosomes from Controls (CDR = 0 and MMSE −) and individuals with dementia (CDR ≥ 1 and MMSE +) from UA-cohort (**a**, **d**), and AD clinically diagnosed cases from UA-cohort (**b**, **e**) or UMG-cohort (**c**, **f**). For WB, each point represents the relative densitometry ratio, and for ELISA, it represents the mean concentration value obtained for each individual. The solid horizontal line shows mean, and error bars indicates standard deviations. Abbreviations: AD, Alzheimer’s disease; C, Controls; CDR, Clinical Dementia Rate; MMSE, Mini-Mental State Examination. *p ≤ 0.05

To validate the results obtained through MS, the levels of AACT and C4BPα were assessed in exosomal-enriched samples isolated with ExoQ, from both Controls and individuals with dementia, including AD cases, from the UA-cohort and the UMG-cohort. The UA-cohort study group included samples from sex- and aged-matched Controls (CDR = 0 and MMSE − , *n* = 32) and individuals with dementia (CDR ≥ 1 and MMSE + , *n* = 32). This dementia group comprises 10 patients that scored CDR = 1 and MMSE + , 22 patients that scored CDR = 2 and 3 and MMSE + and 9 clinically diagnosed AD cases, of which one scored CDR = 1, and was also included in the analyses. The UMG-cohort study group included 12 AD cases, characterized by neuropsychological testing and CSF biomarkers and/or imaging analysis, and respective aged-matched Controls. Serum-derived putative exosomal biomarkers were validated using WB analysis and ELISA in both cohorts. For AACT, WB analysis showed a tendency for an increase in dementia cases, for the UA-cohort. The mean exosomal levels of AACT were 1.29 ± 0.67 for Controls and 1.65 ± 1.05 for CDR ≥ 1 and MMSE + (Fig. 7a). Regarding AD cases, this tendency was also observed, comparatively to the respective sex- and age-matched Controls, although no statistical significance was evident for the UA-cohort (1.92 ± 0.81 vs 2.14 ± 1.71, respectively) (Fig. 7b). However, significant differences were observed for the UMG-cohort, where Controls had lower AACT levels (1.26 ± 0.55) when compared to the AD group (2.12 ± 1.29) (*p* ≤ 0.05) (Fig. 7c). In accordance, ELISA assays revealed likewise a tendency for increased mean levels of AACT in CDR ≥ 1 and MMSE + (11336 ± 2532 pg/mL vs 12228 ± 3274 pg/mL) (Fig. 7d), and AD groups from both UA-cohort (12184 ± 3280 pg/mL vs 13948 ± 4728 pg/mL) (Fig. 7e) and UMG-cohort (10703 ± 3114 pg/mL vs 12035 ± 4247 pg/mL) (Fig. 7f), comparatively to the Control groups. As expected, in accordance with the MS analysis results, an opposite pattern was observed for the C4BPα, for both WB and ELISA assays. Comparatively to the Controls, a tendency to lower C4BPα exosomal levels in the CDR ≥ 1 and MMSE + group (1.01 ± 0.38 vs 0.86 ± 0.24) was obtained by WB analysis (Fig. 8a). When considering only the Controls and respective AD cases, C4BPα levels also tend to decrease although with no significant differences (0.94 ± 0.19 and 0.78 ± 0.19) (Fig. 8b). Consistently, a tendency for decreased levels of C4BPα in AD group (1.79 ± 0.75) when compared with Controls (1.72 ± 0.64) was likewise observed for the UMG-cohort (Fig. 8c). Regarding C4BPα ELISA assays, and comparing against respective Controls, decreased mean levels were obtained for CDR ≥ 1 and MMSE + (19.62 ± 5.41 ng/mL vs 18.09 ± 5.17 ng/mL) (Fig. 8d), and for ADs from the UA-cohort (18.77 ± 4.15 ng/mL vs 17.95 ± 6.15 ng/mL) (Fig. 8e). For disease cases from the UMG-cohort, this decrease reached statistical significance (20.13 ± 5.24 ng/mL vs 16.51 ± 2.72 ng/mL) (*p* ≤ 0.05) (Fig. 8f).

## Discussion

AD blood-based biomarker candidates offer less invasive, cheaper and easier alternatives as a first screening tool to set up in clinical settings, or as complementary tools compared to the currently validated biomarker triplet in CSF and imaging approaches. Exosomes have been described as important players in AD pathogenesis, and these EVs can be isolated efficiently from blood, thus constituting important resources in the diagnosis of this pathology. In this work, two distinct methods (precipitation- and column-based) were employed to isolate serum-derived EVs, with exosome-like characteristics, from Controls and AD cases, and their proteomes were derived through MS and subsequently characterized. Although the size and morphology of the EVs obtained were compatible with the expected exosome features, differences in the particle size distribution and in the number of particles between Controls and AD cases were found for ExoS. Previous studies have reported a tendency for a decreased number of particles in AD cases when compared to Control individuals [14]. Despite that, no significant fluctuations were detected in the number of proteins identified through MS, for exosomes isolated from Controls and AD cases. Further, Control samples from ExoS had a significantly higher particle concentration than Control samples from ExoQ. This may be explained by the different nature of exosome isolation methodologies which can result in different EV subpopulations and yields, and consequently, in distinct exosome proteomes. In general, ExoQ rendered in a higher number of proteins when compared to the ExoS method, which was not surprising since the latter is a column-based approach, and thus more selective. GO analysis revealed that the top 5 biological processes and functions were similar among Controls and disease cases and also between kits. Nonetheless, differences in the proteomes and the global GO analysis were found and this should be addressed in the future, since distinct profiles may be relevant when choosing exosome isolation approaches.

PLS analysis revealed that ExoQ had a higher disease discriminatory power than ExoS, and for this reason data obtained with this kit was pursued in more detail. A set of 9 proteins presented significantly different abundance levels between Controls and ADs. APOC3, APOH, C4BPα, CO3 and KV230 were significantly decreased in ADs, whereas AACT Isoform 1, CO9, IGHM Isoform 2 and K2C6A were increased in the exosomes of ADs. Except for IGHM Isoform 2, K2C6A and KV230, all other proteins have previously been linked to AD, and could present disease discriminatory potential, as presented in Table 2. Indeed, both complements C3 and C5b-C9 have already been tested in plasma astrocyte-derived exosomes [17, 18]. From all identified targets, AACT and C4BPα were the candidates chosen for follow up studies. AACT is an interesting target since it binds Aβ, is found in early stages of senile plaques and promotes its deposition and can induce tau phosphorylation, and its levels have been correlated with cognitive test performance [37–41]. C4BPα is another relevant candidate since it binds the Aβ peptide and can also be found in senile plaques. In addition, it was shown that C4BPα limits the complement activation by Aβ and/or death cells in AD brains, possibly protecting the neuronal environment from immune activation [33]. Moreover, a previous bioinformatic analysis by our group [30] identified both AACT and C4BPα as Aβ-binding proteins in the exosomal proteome, constructed by the overlap of serum-, plasma- and CSF-exosomal proteomes available in databases, namely EXOCARTA, Vesiclepedia or EVpedia. Taken together, we hypothesized that AACT and C4BPα could represent putative AD exosomal biomarker candidates, and thus both markers were tested in serum-derived exosomes from Control and AD cases. Western blot and ELISA approaches were employed to validate AACT and C4BPα as putative AD diagnostic tools. These antibody-based analyses confirmed that the candidate biomarkers, AACT and C4BPα, exhibit variation patterns in agreement with MS results, although significant differences were only found when comparing Controls versus AD cases from the UMG-cohort. This is perhaps not surprising given that the latter was highly characterized by a battery of cognitive and molecular tests. The levels of these proteins have been previously addressed in AD using distinct biofluids (Table 2). Nonetheless, some inconsistencies have been reported, which may relate with the number of individuals enrolled in the study and the distinct techniques used to monitor protein levels. Further, it cannot be excluded that these candidates might also represent early or late-stage biomarkers that alter with disease progression. Indeed, significant correlations were found between AACT exosomal concentrations and MMSE or CDR scores reflecting changes with the cognitive alterations (data not shown). Whether these exosomal biomarkers represent potential candidates for AD diagnosis or general biomarkers for dementia, discriminating the level of cognitive decline, needs to be further validated in a higher number of samples. It would also be interesting to evaluate the levels of these candidates in other neuropathologies, to assess the potential of these two candidates in discriminating AD from other forms of dementia.

## Conclusions

Novel AD diagnostic markers derived from peripheral biofluids, like blood, are urgently needed. Blood-derived exosomes have recently arisen as a novel source of disease biomarkers. Data presented here identifies new exosome putative targets that could distinguish AD cases from Controls. Unravelling the exosome proteome in AD provided a relevant source of blood-based biomarker candidates, easier to implement in clinical practice, which may represent a widely available tool to assist in AD and/or dementia screening.

## Supplementary Information

Below is the link to the electronic supplementary material.

Supplementary file1 (DOCX 255 KB)

## Data Availability

Data generated or analysed during this study are included in this published article and its supplementary information files.

## Abbreviations

AACT: Alpha-1-antichymotrypsin
AD: Alzheimer’s disease
Aβ: Amyloid-beta peptide
C4BPα: C4b-binding protein alpha chain
CDR: Clinical Dementia Rating
CSF: Cerebrospinal fluid
ELISA: Enzyme-linked immunosorbent assay
ExoQ: ExoQuick
ExoS: ExoSpin
EVs: Extracellular vesicles
GO: Gene Ontology
MMSE: Mini-Mental State Examination
MS: Mass spectrometry
NTA: Nanoparticle tracking analysis
PLS: Partial least square
TEM: Transmission electron microscopy
WB: Western blot
