# Supplementary material for: Novel Exosome Biomarker Candidates for Alzheimer’s Disease Unravelled Through Mass Spectrometry Analysis
Source: Mol Neurobiol. 2022 Feb 25;59(5):2838–54. doi: 10.1007/s12035-022-02762-1 (PMC9016047; doi:10.1007/s12035-022-02762-1)
Supplement: Supplementary file 1 — Supplementary file1 (DOCX 255 KB) [file 12035_2022_2762_MOESM1_ESM.docx]

**Online Resources – Supplementary information**

**Molecular Neurobiology**

**Novel exosome biomarker candidates for Alzheimer´s disease unraveled through Mass Spectrometry analysis**

Tânia Soares Martins^1^, Rui Marçalo^1^, Cristóvão B. da Cruz e Silva^2^, Dário Trindade^1^, José Catita^3,4^, Francisco Amado^5^, Tânia Melo^5^, Ilka Martins Rosa^1^, Jonathan Vogelgsang^6,7^, Jens Wiltfang^1,6,8^, Odete A. B. da Cruz e Silva^1^ and Ana Gabriela Henriques^1*^

*^1^Neuroscience and Signalling Group, Department of Medical Sciences, Institute of Biomedicine (iBiMED), University of Aveiro (UA), 3810-193 Aveiro, Portugal*

*^2^Laboratory of Instrumentation and Experimental Particle Physics-LIP, Av. Elias Garcia 14-1º, 1000-149 Lisboa, Portugal*

*^3^CEBIMED—Faculty of Health Sciences; University Fernando Pessoa, 4249-004 Porto, Portugal*

*^4^Paralab SA, 4420-437 Gondomar, Portugal*

*^5^Department of Chemistry, QOPNA (Organic Chemistry Natural and Agrofood Products and LAVQ REQUIMTE), University of Aveiro, 3810-193 Aveiro, Portugal*

*^6^Department of Psychiatry and Psychotherapy, University Medical Center Goettingen (UMG), Georg-August University, Von-Siebold-Str. 5, 37075 Goettingen, Germany*

*^7^Translational Neuroscience Laboratory, McLean Hospital, Harvard Medical School, Belmont, MA 02478, USA*

*^8^German Center for Neurodegenerative Diseases (DZNE), Von-Siebold-Str. 3a, 37075 Goettingen, Germany*

*Corresponding author: aghenriques@ua.pt

**Corresponding author:** Ana Gabriela Henriques, Neuroscience and Signalling Group, Institute of Biomedicine (iBiMED), Department of Medical Sciences, University of Aveiro, 3810-193 Aveiro, Portugal. Tel.: +351 234 370 200; Fax: +351 234 372 587; E-mail: aghenriques@ua.pt.

**Supplementary Figures**

**Supplementary figure 1**

**
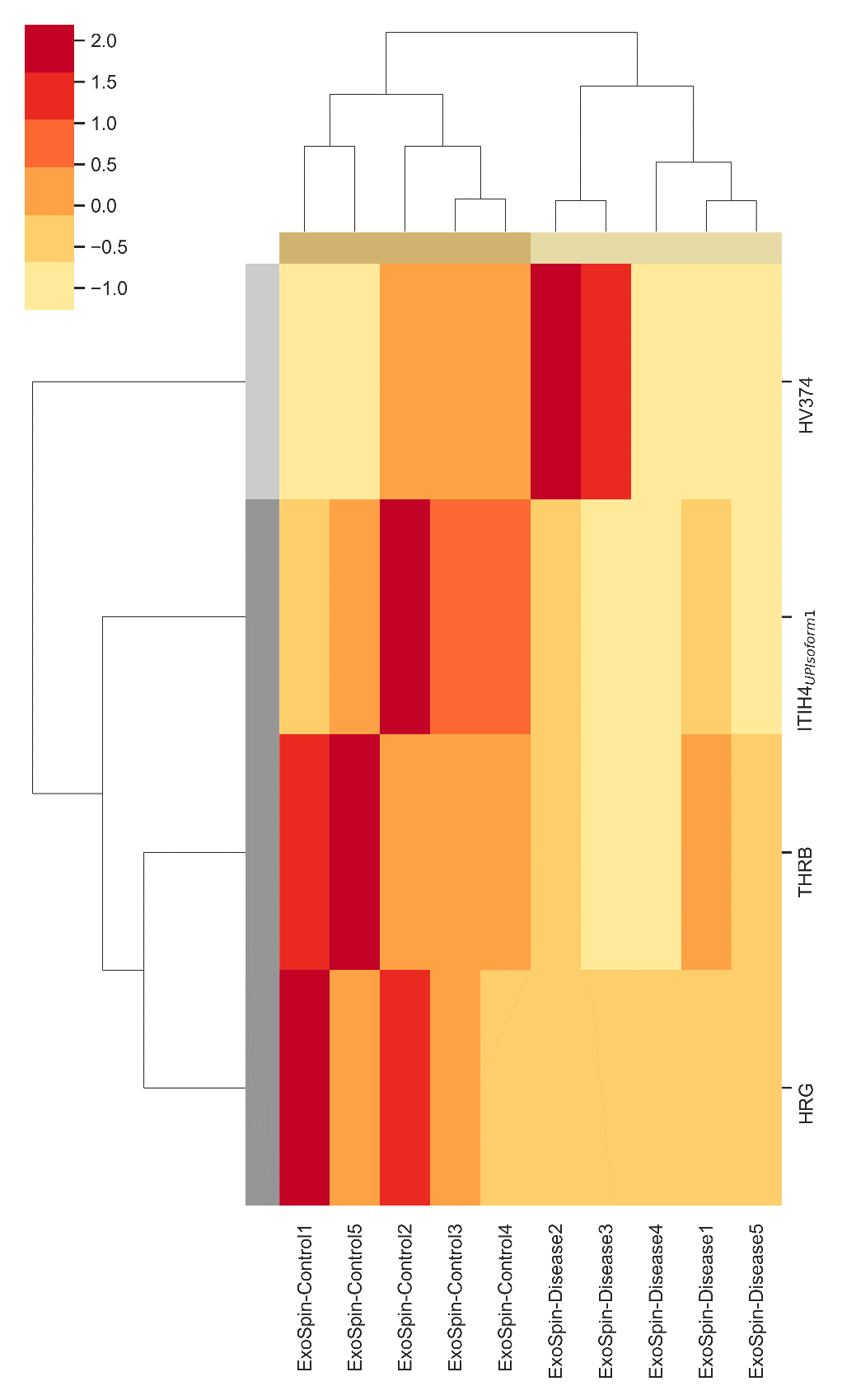
**

**Supplementary figure 1.** **Heatmap of exosomal proteome abundance changes in disease using ExoS.** Heatmaps were constructed for the significant different expressed proteins in AD vs Controls. Differences were determined using Welch’s t-test and 95% confidence level was considered. The bars on the top of heatmaps show the kits category. Red represents higher abundance and light yellow represents lower abundance levels. UniProt protein names and isoforms are indicated at the right. Abbreviations: AD, Alzheimer’s disease; C, Controls; ExoS, ExoSpin; UP, UniProt.

**Supplementary figure 2**

**
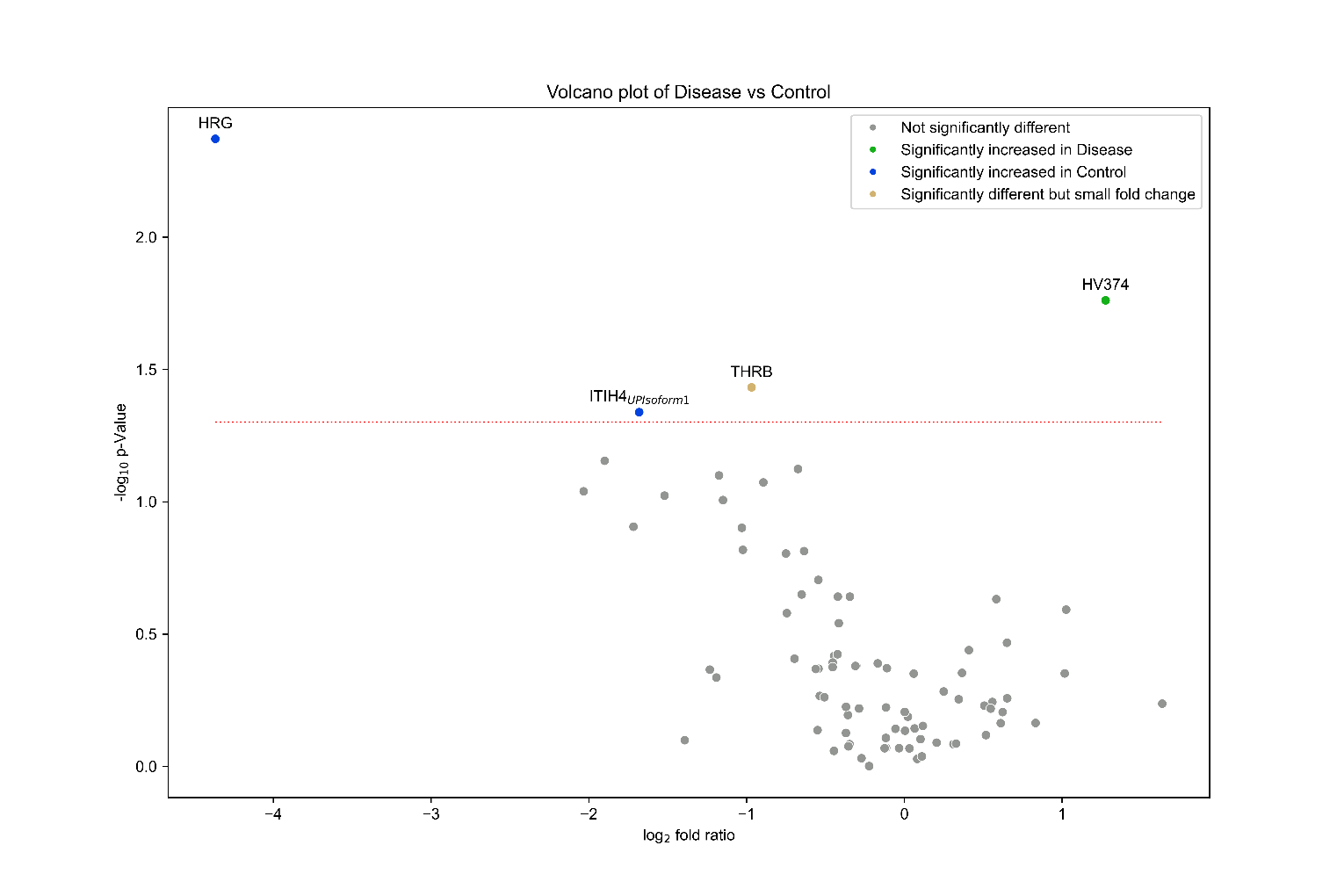
**

**Supplementary figure 2. Volcano plot of significant different exosomal proteins in AD cases versus Controls.** Exosomes were isolated using ExoS. The dashed red line indicates the p-value threshold of 0.05.

**Supplementary table 1. ExoQ exosomal proteome identified through MS**. The proteins included were found in at least one individual either from Controls or Disease group.

| **ExoQ - Exosomal proteome** | | |
| --- | --- | --- |
| **UniProt ID** | **Gene names** | **Protein names** |
| P04217 | A1BG | Alpha-1B-glycoprotein |
| P01023 | A2M | Alpha-2-macroglobulin, Alpha-2-M |
| P61221 | ABCE1 | ATP-binding cassette sub-family E member 1 |
| Q15057 | ACAP2 | Arf-GAP with coiled-coil |
| Q4AC99 | ACCSL | Probable inactive 1-aminocyclopropane-1-carboxylate synthase-like protein 2 |
| P60709 | ACTB | Actin, cytoplasmic 1 |
| Q562R1 | ACTBL2 | Beta-actin-like protein 2 |
| P51816 | AFF2 | AF4/FMR2 family member 2 |
| P43652 | AFM | Afamin |
| Q96MI9-1 | AGBL1 | Cytosolic carboxypeptidase 4 |
| P01019 | AGT | Angiotensinogen |
| P02765 | AHSG | Alpha-2-HS-glycoprotein |
| Q53H80 | AKIRIN2 | Akirin-2 |
| P02768-1; P02768-3 | ALB | Serum albumin |
| P02760 | AMBP | Protein AMBP |
| P07355-2 | ANXA2 | Annexin A2 |
| P02743 | APCS | Serum amyloid P-component |
| P02647 | APOA1 | Apolipoprotein A-I |
| P02652 | APOA2 | Apolipoprotein A-II |
| P06727 | APOA4 | Apolipoprotein A-IV |
| P04114 | APOB | Apolipoprotein B receptor |
| P02656 | APOC3 | Apolipoprotein C-III, Apo-CIII, ApoC-III |
| P05090 | APOD | Apolipoprotein D |
| P02649 | APOE | Apolipoprotein E, Apo-E |
| P02749 | APOH | Beta-2-glycoprotein 1 |
| O14791-2 | APOL1 | Apolipoprotein L1 |
| O95445-1 | APOM | Apolipoprotein M |
| P31939 | ATIC | Bifunctional purine biosynthesis protein PURH |
| P25311 | AZGP1 | Zinc-alpha-2-glycoprotein |
| Q8WY36-3 | BBX | HMG box transcription factor BBX |
| A1A5D9 | BICDL2 | BICD family-like cargo adapter 2 |
| Q8N1H7 | C14orf39 | Protein SIX6OS1 |
| P02745 | C1QA | Complement C1q subcomponent subunit A |
| P02746 | C1QB | Complement C1q subcomponent subunit B |
| P02747 | C1QC | Complement C1q subcomponent subunit C |
| P00736 | C1R | Complement C1r subcomponent |
| P09871 | C1S | Complement C1s subcomponent |
| P06681-1 | C2 | Complement C2 |
| P01024 | C3 | Complement C3 |
| P0C0L4-1 | C4A | Complement C4-A |
| P0C0L5 | C4B | Complement C4-B |
| P04003 | C4BPA | C4b-binding protein alpha chain |
| P20851 | C4BPB | C4b-binding protein beta chain |
| P01031 | C5 | Complement C5 |
| P13671 | C6 | Complement component C6 |
| P10643 | C7 | Complement component C7 |
| P07357 | C8A | Complement component C8 alpha chain |
| P07358 | C8B | Complement component C8 beta chain |
| P07360 | C8G | Complement component C8 gamma chain |
| P02748 | C9 | Complement component C9 |
| Q6ZRK6-1 | CCDC73 | Coiled-coil domain-containing protein 73 |
| Q9NVE4 | CCDC87 | Coiled-coil domain-containing protein 87 |
| O43866 | CD5L | CD5 antigen-like |
| Q96N23 | CFAP54 | Cilia- and flagella-associated protein 54 |
| Q96MR6-1 | CFAP57 | Cilia- and flagella-associated protein 57 |
| P00751-1 | CFB | Complement factor B |
| P08603-1 | CFH | Complement factor H |
| Q03591 | CFHR1 | Complement factor H-related protein 1 |
| P36980-1 | CFHR2 | Complement factor H-related protein 2 |
| Q92496 | CFHR4 | Complement factor H-related protein 4 |
| P05156 | CFI | Complement factor I |
| P27918 | CFP | Properdin |
| Q9P2E5-1 | CHPF2 | Chondroitin sulfate glucuronyltransferase |
| Q6WN34-3 | CHRDL2 | Chordin-like protein 2 |
| P05452 | CLEC3B | Tetranectin |
| P10909-2 | CLU | Clusterin |
| Q96DG6 | CMBL | Carboxymethylenebutenolidase homolog |
| Q96KN2 | CNDP1 | Beta-Ala-His dipeptidase |
| A5YKK6 | CNOT1 | CCR4-NOT transcription complex subunit 1 |
| Q07092-2 | COL16A1 | Collagen alpha-1(XVI) chain |
| P49747 | COMP | Cartilage oligomeric matrix protein |
| P00450 | CP | Ceruloplasmin |
| P22792 | CPN2 | Carboxypeptidase N subunit 2 |
| P02741-1 | CRP | C-reactive protein |
| A4D2H0 | CTAGE15 | cTAGE family member 15 |
| Q9BUQ8 | DDX23 | Probable ATP-dependent RNA helicase DDX23 |
| Q08211 | DHX9 | ATP-dependent RNA helicase A |
| O14490 | DLGAP1 | Disks large-associated protein 1 |
| Q3MIW9 | DPCR1 | Mucin-like protein 3 |
| Q02413 | DSG1 | Desmoglein-1 |
| Q03001-7 | DST | Dystonin |
| Q16610-4 | ECM1 | Extracellular matrix protein 1 |
| Q12805 | EFEMP1 | EGF-containing fibulin-like extracellular matrix protein 1 |
| P21709 | EPHA1 | Ephrin type-A receptor 1 |
| P15036 | ETS2 | Protein C-ets-2 |
| P03951 | F11 | Coagulation factor XI |
| P00748 | F12 | Coagulation factor XII |
| P05160 | F13B | Coagulation factor XIII B chain |
| P00734 | F2 | Prothrombin |
| Q01469 | FABP5 | Fatty acid-binding protein 5 |
| Q96PS1 | FANCD2OS | FANCD2 opposite strand protein |
| P23142; P23142-4 | FBLN1 | Fibulin-1 |
| Q9Y6R7 | FCGBP | IgGFc-binding protein |
| P31995-2 | FCGR2C | Low affinity immunoglobulin gamma Fc region receptor II-c |
| O75636-1 | FCN3 | Ficolin-3 |
| P02751-15 | FN1 | Fibronectin, FN |
| Q5VWT5-1 | FYB2 | FYN-binding protein 2 |
| P02774-3 | GC | Vitamin D-binding protein, DBP |
| Q14161-6 | GIT2 | ARF GTPase-activating protein GIT2 |
| P16278 | GLB1 | Beta-galactosidase |
| P80108 | GPLD1 | Phosphatidylinositol-glycan-specific phospholipase D |
| P22352 | GPX3 | Glutathione peroxidase 3 |
| Q13003 | GRIK3 | Glutamate receptor ionotropic, kainate 3 |
| P06396 | GSN | Gelsolin |
| P62805 | H4C1 | Histone H4 |
| Q14520-1 | HABP2 | Hyaluronan-binding protein 2 |
| P69905 | HBA1 | Hemoglobin subunit alpha |
| P68871 | HBB | Hemoglobin subunit beta |
| P02042 | HBD | Hemoglobin subunit delta |
| P51610-4 | HCFC1 | Host cell factor 1 |
| P00738 | HP | Haptoglobin |
| P00739-2 | HPR | Glycerate dehydrogenase HPR |
| P02790 | HPX | Hemopexin |
| P04196 | HRG | Histidine-rich glycoprotein |
| Q86YZ3 | HRNR | Hornerin |
| Q8TCB0 | IFI44 | Interferon-induced protein 44-like |
| P35858-2 | IGFALS | Insulin-like growth factor-binding protein complex acid labile subunit, ALS |
| P01876 | IGHA1 | Immunoglobulin heavy constant alpha 1 |
| P01859 | IGHG2 | Immunoglobulin heavy constant gamma 2 |
| P01860 | IGHG3 | Immunoglobulin heavy constant gamma 3 |
| P01861 | IGHG4 | Immunoglobulin heavy constant gamma 4 |
| P01871; P01871-2; P04220 | IGHM | Immunoglobulin heavy constant mu |
| A0A0C4DH31 | IGHV1-18 | Immunoglobulin heavy variable 1-18 |
| A0A0B4J2H0 | IGHV1-69D | Immunoglobulin heavy variable 1-69D |
| P23083 | IGHV1OR15-1 | Immunoglobulin heavy variable 1/OR15-1 |
| P01766 | IGHV3-13 | Immunoglobulin heavy variable 3-13 |
| A0A0B4J1V0 | IGHV3-15 | Immunoglobulin heavy variable 3-15 |
| A0A0B4J1V1 | IGHV3-21 | Immunoglobulin heavy variable 3-21 |
| P01764 | IGHV3-23 | Immunoglobulin heavy variable 3-23 |
| P01780 | IGHV3-7 | Immunoglobulin heavy variable 3-7 |
| A0A0B4J1Y9 | IGHV3-72 | Immunoglobulin heavy variable 3-72 |
| A0A0B4J1X5 | IGHV3-74 | Immunoglobulin heavy variable 3-74 |
| P01824 | IGHV4-39 | Immunoglobulin heavy variable 4-39 |
| A0A0B4J1U7 | IGHV6-1 | Immunoglobulin heavy variable 6-1 |
| P01834 | IGKC | Immunoglobulin kappa constant |
| P04430 | IGKV1-16 | Immunoglobulin kappa variable 1-16 |
| P01599 | IGKV1-17 | Immunoglobulin kappa variable 1-17 |
| A0A075B6S5 | IGKV1-27 | Immunoglobulin kappa variable 1-27 |
| P01602 | IGKV1-5 | Immunoglobulin kappa variable 1-5 |
| A0A0C4DH72 | IGKV1-6 | Immunoglobulin kappa variable 1-6 |
| A0A0B4J2D9 | IGKV1D-13 | Immunoglobulin kappa variable 1D-13 |
| P01594 | IGKV1D-33 | Immunoglobulin kappa variable 1D-33 |
| A0A0C4DH68 | IGKV2-24 | Immunoglobulin kappa variable 2-24 |
| P06310 | IGKV2-30 | Immunoglobulin kappa variable 2-30 |
| P01614 | IGKV2D-40 | Immunoglobulin kappa variable 2D-40 |
| P04433 | IGKV3-11 | Immunoglobulin kappa variable 3-11 |
| P01624 | IGKV3-15 | Immunoglobulin kappa variable 3-15 |
| P01619 | IGKV3-20 | Immunoglobulin kappa variable 3-20 |
| A0A0A0MRZ8 | IGKV3D-11 | Immunoglobulin kappa variable 3D-11 |
| A0A087WSY6 | IGKV3D-15 | Immunoglobulin kappa variable 3D-15 |
| A0A0C4DH25 | IGKV3D-20 | Immunoglobulin kappa variable 3D-20 |
| A0A0C4DH55 | IGKV3D-7 | Immunoglobulin kappa variable 3D-7 |
| P06312 | IGKV4-1 | Immunoglobulin kappa variable 4-1 |
| P0DOY2 | IGLC2 | Immunoglobulin lambda constant 2 |
| P0DOY3 | IGLC3 | Immunoglobulin lambda constant 3 |
| P0CF74 | IGLC6 | Immunoglobulin lambda constant 6 |
| A0M8Q6 | IGLC7 | Immunoglobulin lambda constant 7 |
| P15814 | IGLL1 | Immunoglobulin lambda-like polypeptide 1 |
| A0A0B4J1U3 | IGLV1-36 | Immunoglobulin lambda variable 1-36 |
| P01703 | IGLV1-40 | Immunoglobulin lambda variable 1-40 |
| P01699 | IGLV1-44 | Immunoglobulin lambda variable 1-44 |
| P01700 | IGLV1-47 | Immunoglobulin lambda variable 1-47 |
| P01701 | IGLV1-51 | Immunoglobulin lambda variable 1-51 |
| P01706 | IGLV2-11 | Immunoglobulin lambda variable 2-11 |
| P01704 | IGLV2-14 | Immunoglobulin lambda variable 2-14 |
| A0A075B6J9 | IGLV2-18 | Immunoglobulin lambda variable 2-18 |
| P01705 | IGLV2-23 | Immunoglobulin lambda variable 2-23 |
| P01709 | IGLV2-8 | Immunoglobulin lambda variable 2-8 |
| A0A075B6K4 | IGLV3-10 | Immunoglobulin lambda variable 3-10 |
| P01714 | IGLV3-19 | Immunoglobulin lambda variable 3-19 |
| P80748 | IGLV3-21 | Immunoglobulin lambda variable 3-21 |
| P01717 | IGLV3-25 | Immunoglobulin lambda variable 3-25 |
| A0A075B6I9 | IGLV7-46 | Immunoglobulin lambda variable 7-46 |
| A0A075B6I0 | IGLV8-61 | Immunoglobulin lambda variable 8-61 |
| A6NCM1 | IQCA1L | IQ and AAA domain-containing protein 1-like |
| P38570 | ITGAE | Integrin alpha-E |
| P19827-1 | ITIH1 | Inter-alpha-trypsin inhibitor heavy chain H1 |
| P19823 | ITIH2 | Inter-alpha-trypsin inhibitor heavy chain H2 |
| Q06033-1 | ITIH3 | Inter-alpha-trypsin inhibitor heavy chain H3 |
| Q14624-1 | ITIH4 | Inter-alpha-trypsin inhibitor heavy chain H4 |
| P01591 | JCHAIN | Immunoglobulin J chain |
| Q12756-2 | KIF1A | Kinesin-like protein KIF1A |
| P03952 | KLKB1 | Plasma kallikrein |
| A4D1S0-2 | KLRG2 | Killer cell lectin-like receptor subfamily G member 2 |
| P01042 | KNG1 | Kininogen-1 |
| Q5T749 | KPRP | Keratinocyte proline-rich protein |
| P04264 | KRT1 | Keratin, type II cytoskeletal 1 |
| P13645 | KRT10 | Keratin, type I cytoskeletal 10 |
| P02533 | KRT14 | Keratin, type I cytoskeletal 14 |
| P08779 | KRT16 | Keratin, type I cytoskeletal 16 |
| Q04695 | KRT17 | Keratin, type I cytoskeletal 17 |
| P35908 | KRT2 | Keratin, type II cytoskeletal 2 epidermal |
| Q2M2I5 | KRT24 | Keratin, type I cytoskeletal 24 |
| P12035 | KRT3 | Keratin, type II cytoskeletal 3 |
| P13647 | KRT5 | Keratin, type II cytoskeletal 5 |
| P02538 | KRT6A | Keratin, type II cytoskeletal 6A |
| P04259 | KRT6B | Keratin, type II cytoskeletal 6B |
| P48668 | KRT6C | Keratin, type II cytoskeletal 6C |
| P78386 | KRT85 | Keratin, type II cuticular Hb5 |
| P35527 | KRT9 | Keratin, type I cytoskeletal 9 |
| Q13094 | LCP2 | Lymphocyte cytosolic protein 2 |
| Q08380 | LGALS3BP | Galectin-3-binding protein |
| P47929 | LGALS7 | Galectin-7 |
| P08519 | LPA | Lysophosphatidic acid receptor 1 |
| P02750 | LRG1 | Rho-GTPase-activating protein LRG1 |
| Q92585 | MAML1 | Mastermind-like protein 1 |
| P48740-2 | MASP1 | Mannan-binding lectin serine protease 1 |
| O00187-1 | MASP2 | Mannan-binding lectin serine protease 2 |
| Q14566 | MCM6 | DNA replication licensing factor MCM6 |
| Q9NXB0-1 | MKS1 | Meckel syndrome type 1 protein |
| P26927 | MST1 | Hepatocyte growth factor-like protein |
| P03897 | ND3 | NADH-ubiquinone oxidoreductase chain 3 |
| Q16236 | NFE2L2 | Nuclear factor erythroid 2-related factor 2 |
| Q9H0P0-4 | NT5C3A | Cytosolic 5'-nucleotidase 3A |
| Q6IFN5 | OR7E24 | Olfactory receptor 7E24 |
| P02763 | ORM1 | Protein ORM1 |
| P11498 | PC | Pyruvate carboxylase, mitochondrial |
| P02776 | PF4 | Platelet factor 4, PF-4 |
| Q96PD5-2 | PGLYRP2 | N-acetylmuramoyl-L-alanine amidase |
| Q9Y2I7-1 | PIKFYVE | 1-phosphatidylinositol 3-phosphate 5-kinase |
| Q15149-1 | PLEC | Plectin |
| P00747 | PLG | Plasminogen |
| O43660-1 | PLRG1 | Pleiotropic regulator 1 |
| O75417 | POLQ | DNA polymerase theta |
| P27169 | PON1 | Serum paraoxonase/arylesterase 1 |
| A5A3E0 | POTEF | POTE ankyrin domain family member F |
| Q07869 | PPARA | Peroxisome proliferator-activated receptor alpha |
| P02775 | PPBP | Platelet basic protein, PBP |
| O60237-3 | PPP1R12B | Protein phosphatase 1 regulatory subunit 12B |
| P78527 | PRKDC | DNA-dependent protein kinase catalytic subunit |
| P07225 | PROS1 | Vitamin K-dependent protein S |
| P07477 | PRSS1 | Trypsin-1 |
| P29074 | PTPN4 | Tyrosine-protein phosphatase non-receptor type 4 |
| P20742 | PZP | Pregnancy zone protein |
| Q9Y2K5-1 | R3HDM2 | R3H domain-containing protein 2 |
| Q8TEU7-4 | RAPGEF6 | Rap guanine nucleotide exchange factor 6 |
| P02753 | RBP4 | Retinol-binding protein 4 |
| Q96MK2-1 | RIPOR3 | RIPOR family member 3 |
| Q5VT52 | RPRD2 | Regulation of nuclear pre-mRNA domain-containing protein 2 |
| P62979 | RPS27A | Ubiquitin-40S ribosomal protein S27a |
| P05109 | S100A8 | Protein S100-A8 |
| P0DJI8 | SAA1 | Serum amyloid A-1 protein |
| P35542 | SAA4 | Serum amyloid A-4 protein |
| Q9UI33-1 | SCN11A | Sodium channel protein type 11 subunit alpha |
| P31431-1 | SDC4 | Syndecan-4 |
| P01009-1 | SERPINA1 | Alpha-1-antitrypsin |
| P01011-1 | SERPINA3 | Alpha-1-antichymotrypsin |
| P05154 | SERPINA5 | Plasma serine protease inhibitor |
| P01008 | SERPINC1 | Antithrombin-III |
| P05546 | SERPIND1 | Heparin cofactor 2 |
| P36955 | SERPINF1 | Pigment epithelium-derived factor, PEDF |
| P08697-1 | SERPINF2 | Alpha-2-antiplasmin |
| P05155-3 | SERPING1 | Plasma protease C1 inhibitor |
| P31947-1 | SFN | 14-3-3 protein sigma |
| P78539-1 | SRPX | Sushi repeat-containing protein SRPX |
| Q9BXU1-1 | STK31 | Serine/threonine-protein kinase 31 |
| P02787 | TF | Serotransferrin, Transferrin |
| P07996 | THBS1 | Thrombospondin-1 |
| P35443 | THBS4 | Thrombospondin-4 |
| Q9UDY2-7 | TJP2 | Tight junction protein ZO-2 |
| Q14166 | TTLL12 | Tubulin-tyrosine ligase-like protein 12 |
| Q8WZ42-12 | TTN | TTN |
| P02766 | TTR | Transthyretin |
| Q6ZT12-4 | UBR3 | E3 ubiquitin-protein ligase UBR3 |
| P04004 | VTN | Vitronectin |
| P04275 | VWF | von Willebrand factor |
| Q9UK11 | ZNF223 | Zinc finger protein 223 |
| Q6NX45 | ZNF774 | Zinc finger protein 774 |
| P0DOX2 | - | Immunoglobulin alpha-2 heavy chain |
| P0DOX3 | - | Immunoglobulin delta heavy chain |
| P0DOX4 | - | Immunoglobulin epsilon heavy chain |
| P0DOX5 | - | Immunoglobulin gamma-1 heavy chain |
| P0DOX6 | - | Immunoglobulin mu heavy chain |
| P0DOX7 | - | Immunoglobulin kappa light chain |
| P0DOX8 | - | Immunoglobulin lambda-1 light chain |

**Supplementary table 2. ExoS exosomal proteome identified through MS**. The proteins included were found in at least one individual either from Controls or Disease group.

| **ExoS - Exosomal proteome** | | |
| --- | --- | --- |
| **UniProt ID** | **Gene names** | **Protein names** |
| P04217 | A1BG | Alpha-1B-glycoprotein |
| P01023 | A2M | Alpha-2-macroglobulin |
| Q9UHI8 | ADAMTS1 | A disintegrin and metalloproteinase with thrombospondin motifs 1 |
| P01019 | AGT | Angiotensinogen |
| P02768-1 | ALB | Serum albumin |
| P02760 | AMBP | Protein AMBP |
| P02743 | APCS | Serum amyloid P-component |
| P02647 | APOA1 | Apolipoprotein A-I |
| P06727 | APOA4 | Apolipoprotein A-IV |
| P04114 | APOB | Apolipoprotein B-100 |
| P05090 | APOD | Apolipoprotein D |
| P02649 | APOE | Apolipoprotein E |
| P02749 | APOH | Beta-2-glycoprotein 1 |
| O14791-2 | APOL1 | Apolipoprotein L1 |
| O95445-1 | APOM | Apolipoprotein M |
| P31939 | ATIC | Bifunctional purine biosynthesis protein PURH |
| P25311 | AZGP1 | Zinc-alpha-2-glycoprotein |
| P02745 | C1QA | Complement C1q subcomponent subunit A |
| P02747 | C1QC | Complement C1q subcomponent subunit C |
| P00736 | C1R | Complement receptor type 1 |
| P09871 | C1S | Complement C1s subcomponent |
| P06681-1 | C2 | Complement C2 |
| P01024 | C3 | Complement C3 |
| P0C0L4-1 | C4A | Complement C4-A |
| P0C0L5 | C4B | Complement C4-B |
| P04003 | C4BPA | C4b-binding protein alpha chain |
| P20851 | C4BPB | C4b-binding protein beta chain |
| P01031 | C5 | Complement C5 |
| P13671 | C6 | Complement component C6 |
| P10643 | C7 | Complement component C7 |
| P07358 | C8B | Complement component C8 beta chain |
| P07360 | C8G | Complement component C8 gamma chain |
| P02748 | C9 | Complement component C9 |
| O43866 | CD5L | CD5 antigen-like |
| P00751-1 | CFB | Complement factor B |
| P08603-1 | CFH | Complement factor H |
| Q03591 | CFHR1 | Complement factor H-related protein 1 |
| P36980-1 | CFHR2 | Complement factor H-related protein 2 |
| P27918 | CFP | Properdin |
| Q8TDI0 | CHD5 | Chromodomain-helicase-DNA-binding protein 5 |
| P05452 | CLEC3B | Tetranectin |
| Q00610-1 | CLTC | Clathrin heavy chain 1 |
| P10909-2 | CLU | Clusterin |
| P00450 | CP | Ceruloplasmin |
| P02741-1 | CRP | Cysteine-rich protein 1 |
| Q08211 | DHX9 | ATP-dependent RNA helicase A |
| Q02413 | DSG1 | Desmoglein-1 |
| Q16610-4 | ECM1 | Extracellular matrix protein 1 |
| Q13822-2 | ENPP2 | Ectonucleotide pyrophosphatase/phosphodiesterase family member 2 |
| O75354-3 | ENTPD6 | Ectonucleoside triphosphate diphosphohydrolase 6 |
| P00748 | F12 | Coagulation factor XII |
| P05160 | F13B | Coagulation factor XIII B chain |
| P00734 | F2 | Prothrombin |
| P0CG43 | FAM157C | Putative protein FAM157C |
| P23142 | FBLN1 | Fibulin-1 |
| Q9Y6R7 | FCGBP | IgGFc-binding protein |
| O75636-1 | FCN3 | Ficolin-3 |
| Q8N539-1 | FIBCD1 | Fibrinogen C domain-containing protein 1 |
| P02751-15 | FN1 | Fibronectin |
| P02774-3 | GC | Vitamin D-binding protein |
| Q9NR23 | GDF3 | Growth/differentiation factor 3 |
| P06396 | GSN | Gelsolin |
| P62805 | H4C1 | Histone H4 |
| P69905 | HBA1 | Hemoglobin subunit alpha |
| P68871 | HBB | Hemoglobin subunit beta |
| P02042 | HBD | Hemoglobin subunit delta |
| P00738 | HP | Haptoglobin |
| P00739-2 | HPR | Heparanase |
| P02790 | HPX | Hemopexin |
| P04196 | HRG | Histidine-rich glycoprotein |
| Q86YZ3 | HRNR | Hornerin |
| P04792 | HSPB1 | Heat shock protein beta-1 |
| P35858-2 | IGFALS | Insulin-like growth factor-binding protein complex acid labile subunit |
| P01876 | IGHA1 | Immunoglobulin heavy constant alpha 1 |
| P01857 | IGHG1 | Immunoglobulin heavy constant gamma 1 |
| P01859 | IGHG2 | Immunoglobulin heavy constant gamma 2 |
| P01860 | IGHG3 | Immunoglobulin heavy constant gamma 3 |
| P01861 | IGHG4 | Immunoglobulin heavy constant gamma 4 |
| P01871-2 | IGHM | Immunoglobulin heavy constant mu |
| A0A0C4DH31 | IGHV1-18 | Immunoglobulin heavy variable 1-18 |
| P23083 | IGHV1OR15-1 | Immunoglobulin heavy variable 1/OR15-1 |
| A0A0B4J1V0 | IGHV3-15 | Immunoglobulin heavy variable 3-15 |
| P01764 | IGHV3-23 | Immunoglobulin heavy variable 3-23 |
| A0A0B4J1X5 | IGHV3-74 | Immunoglobulin heavy variable 3-74 |
| A0A0B4J1U7 | IGHV6-1 | Immunoglobulin heavy variable 6-1 |
| P01834 | IGKC | Immunoglobulin kappa constant |
| A0A075B6S5 | IGKV1-27 | Immunoglobulin kappa variable 1-27 |
| A0A0C4DH72 | IGKV1-6 | Immunoglobulin kappa variable 1-6 |
| P01594 | IGKV1D-33 | Immunoglobulin kappa variable 1D-33 |
| P06310 | IGKV2-30 | Immunoglobulin kappa variable 2-30 |
| P01614 | IGKV2D-40 | Immunoglobulin kappa variable 2D-40 |
| P01624 | IGKV3-15 | Immunoglobulin kappa variable 3-15 |
| P01619 | IGKV3-20 | Immunoglobulin kappa variable 3-20 |
| A0A0A0MRZ8 | IGKV3D-11 | Immunoglobulin kappa variable 3D-11 |
| A0A0C4DH55 | IGKV3D-7 | Immunoglobulin kappa variable 3D-7 |
| P0DOY2 | IGLC2 | Immunoglobulin lambda constant 2 |
| P0DOY3 | IGLC3 | Immunoglobulin lambda constant 3 |
| P0CF74 | IGLC6 | Immunoglobulin lambda constant 6 |
| A0M8Q6 | IGLC7 | Immunoglobulin lambda constant 7 |
| P15814 | IGLL1 | Immunoglobulin lambda-like polypeptide 1 |
| P01700 | IGLV1-47 | Immunoglobulin lambda variable 1-47 |
| P01701 | IGLV1-51 | Immunoglobulin lambda variable 1-51 |
| P80748 | IGLV3-21 | Immunoglobulin lambda variable 3-21 |
| A0A075B6I9 | IGLV7-46 | Immunoglobulin lambda variable 7-46 |
| A0A075B6I0 | IGLV8-61 | Immunoglobulin lambda variable 8-61 |
| P19827-1 | ITIH1 | Inter-alpha-trypsin inhibitor heavy chain H1 |
| P19823 | ITIH2 | Inter-alpha-trypsin inhibitor heavy chain H2 |
| Q06033-1 | ITIH3 | Inter-alpha-trypsin inhibitor heavy chain H3 |
| Q14624-1; Q14624-2 | ITIH4 | Inter-alpha-trypsin inhibitor heavy chain H4 |
| P01591 | JCHAIN | Immunoglobulin J chain |
| Q5VYK3 | KIAA0368 | Proteasome adapter and scaffold protein ECM29 |
| O60333-3 | KIF1B | Kinesin-like protein KIF1B |
| Q13886 | KLF9 | Krueppel-like factor 9 |
| P06870-1 | KLK1 | Kallikrein-1 |
| P03952 | KLKB1 | Plasma kallikrein |
| P01042; P01042-2 | KNG1 | Kininogen-1 |
| Q5T749 | KPRP | Keratinocyte proline-rich protein |
| P04264 | KRT1 | Keratin, type II cytoskeletal 1 |
| P13645 | KRT10 | Keratin, type I cytoskeletal 10 |
| P02533 | KRT14 | Keratin, type I cytoskeletal 14 |
| P08779 | KRT16 | Keratin, type I cytoskeletal 16 |
| P35908 | KRT2 | Keratin, type II cytoskeletal 2 epidermal |
| P13647 | KRT5 | Keratin, type II cytoskeletal 5 |
| P02538 | KRT6A | Keratin, type II cytoskeletal 6A |
| P04259 | KRT6B | Keratin, type II cytoskeletal 6B |
| P48668 | KRT6C | Keratin, type II cytoskeletal 6C |
| P35527 | KRT9 | Keratin, type I cytoskeletal 9 |
| Q08380 | LGALS3BP | Galectin-3-binding protein |
| P47929 | LGALS7 | Galectin-7 |
| P08519 | LPA | Apolipoprotein(a) |
| Q8WZ04-1 | LRTOMT | Transmembrane O-methyltransferase |
| O00187-1 | MASP2 | Mannan-binding lectin serine protease 2 |
| P25205-2 | MCM3 | DNA replication licensing factor MCM3 |
| Q03111 | MLLT1 | Protein ENL |
| Q86VD1 | MORC1 | MORC family CW-type zinc finger protein 1 |
| B2RTY4-4 | MYO9A | Unconventional myosin-IXa |
| Q8TBE9 | NANP | N-acylneuraminate-9-phosphatase |
| O76041 | NEBL | Nebulette |
| Q9Y2I1-1 | NISCH | Nischarin |
| P02763 | ORM1 | Alpha-1-acid glycoprotein 1 |
| Q15120-2 | PDK3 | [Pyruvate dehydrogenase (acetyl-transferring)] kinase isozyme 3, mitochondrial |
| Q96PD5-2 | PGLYRP2 | N-acetylmuramoyl-L-alanine amidase |
| Q9H5I5-4 | PIEZO2 | Piezo-type mechanosensitive ion channel component 2 |
| Q9Y2I7-1 | PIKFYVE | 1-phosphatidylinositol 3-phosphate 5-kinase |
| Q9UKJ0-3 | PILRB | Paired immunoglobulin-like type 2 receptor beta |
| P00747 | PLG | Plasminogen |
| P27169 | PON1 | Serum paraoxonase/arylesterase 1 |
| A5A3E0 | POTEF | POTE ankyrin domain family member F |
| P02775 | PPBP | Platelet basic protein |
| Q8TF05 | PPP4R1 | Serine/threonine-protein phosphatase 4 regulatory subunit 1 |
| P02810 | PRH1 | Salivary acidic proline-rich phosphoprotein 1/2 |
| P24723 | PRKCH | Protein kinase C eta type |
| P07225 | PROS1 | Vitamin K-dependent protein S |
| P07477 | PRSS1 | Trypsin-1 |
| P29074 | PTPN4 | Tyrosine-protein phosphatase non-receptor type 4 |
| P23467-3 | PTPRB | Receptor-type tyrosine-protein phosphatase beta |
| P20742 | PZP | Pregnancy zone protein |
| Q9BXF6 | RAB11FIP5 | Rab11 family-interacting protein 5 |
| P02753 | RBP4 | Retinol-binding protein 4 |
| Q6NUM9-1 | RETSAT | All-trans-retinol 13,14-reductase |
| O75150 | RNF40 | E3 ubiquitin-protein ligase BRE1B, BRE1-B |
| P0DJI8 | SAA1 | Serum amyloid A-1 protein |
| P35542 | SAA4 | Serum amyloid A-4 protein |
| P01009-1 | SERPINA1 | Alpha-1-antitrypsin |
| P01011-1 | SERPINA3 | Alpha-1-antichymotrypsin |
| P01008 | SERPINC1 | Antithrombin-III |
| P05546 | SERPIND1 | Heparin cofactor 2 |
| P36955 | SERPINF1 | Pigment epithelium-derived factor |
| P04278-1 | SHBG | Sex hormone-binding globulin |
| Q07890 | SOS2 | Son of sevenless homolog 2 |
| P49675 | STAR | Steroidogenic acute regulatory protein, mitochondrial |
| Q7Z7C8-4 | TAF8 | Transcription initiation factor TFIID subunit 8 |
| Q9H6P5-2 | TASP1 | Threonine aspartase 1 |
| P02787 | TF | Serotransferrin, Transferrin |
| P07996 | THBS1 | Thrombospondin-1 |
| P35443 | THBS4 | Thrombospondin-4 |
| Q92545 | TMEM131 | Transmembrane protein 131 |
| Q9ULQ1-3 | TPCN1 | Two pore calcium channel protein 1 |
| P02766 | TTR | Transthyretin |
| Q14146 | URB2 | Unhealthy ribosome biogenesis protein 2 homolog |
| Q5THJ4 | VPS13D | Vacuolar protein sorting-associated protein 13D |
| P04004 | VTN | Vitronectin |
| P04275 | VWF | von Willebrand factor |
| O00401 | WASL | Neural Wiskott-Aldrich syndrome protein |
| P0DOX2 | - | Immunoglobulin alpha-2 heavy chain |
| P0DOX4 | - | Immunoglobulin epsilon heavy chain |
| P0DOX5 | - | Immunoglobulin gamma-1 heavy chain |
| P0DOX6 | - | Immunoglobulin mu heavy chain |
| P0DOX7 | - | Immunoglobulin kappa light chain |
| P0DOX8 | - | Immunoglobulin lambda-1 light chain |

**Supplementary table 3. ExoS and ExoQ common exosomal proteins identified through MS**. Common proteins isolated from the two kits (considering both Controls and AD cases). The proteins included were found in at least one individual.

| **Overlap of proteins identified using ExoQ and ExoS** | | |
| --- | --- | --- |
| **UniProt ID** | **Gene names** | **Protein names** |
| P04217 | A1BG | Alpha-1B-glycoprotein |
| P01023 | A2M | Alpha-2-macroglobulin |
| P01019 | AGT | Angiotensinogen |
| P02768-1 | ALB | Serum albumin |
| P02760 | AMBP | Protein AMBP |
| P02743 | APCS | Serum amyloid P-component |
| P02647 | APOA1 | Apolipoprotein A-I |
| P06727 | APOA4 | Apolipoprotein A-IV |
| P04114 | APOB | Apolipoprotein B-100 |
| P05090 | APOD | Apolipoprotein D |
| P02649 | APOE | Apolipoprotein E |
| P02749 | APOH | Beta-2-glycoprotein 1 |
| O14791-2 | APOL1 | Apolipoprotein L1 |
| O95445-1 | APOM | Apolipoprotein M |
| P31939 | ATIC | Bifunctional purine biosynthesis protein PURH |
| P25311 | AZGP1 | Zinc-alpha-2-glycoprotein |
| P02745 | C1QA | Complement C1q subcomponent subunit A |
| P02747 | C1QC | Complement C1q subcomponent subunit C |
| P00736 | C1R | Complement receptor type 1 |
| P09871 | C1S | Complement C1s subcomponent |
| P06681-1 | C2 | Complement C2 |
| P01024 | C3 | Complement C3 |
| P0C0L4-1 | C4A | Complement C4-A |
| P0C0L5 | C4B | Complement C4-B |
| P04003 | C4BPA | C4b-binding protein alpha chain |
| P20851 | C4BPB | C4b-binding protein beta chain |
| P01031 | C5 | Complement C5 |
| P13671 | C6 | Complement component C6 |
| P10643 | C7 | Complement component C7 |
| P07358 | C8B | Complement component C8 beta chain |
| P07360 | C8G | Complement component C8 gamma chain |
| P02748 | C9 | Complement component C9 |
| O43866 | CD5L | CD5 antigen-like |
| P00751-1 | CFB | Complement factor B |
| P08603-1 | CFH | Complement factor H |
| Q03591 | CFHR1 | Complement factor H-related protein 1 |
| P36980-1 | CFHR2 | Complement factor H-related protein 2 |
| P27918 | CFP | Properdin |
| P05452 | CLEC3B | Tetranectin |
| P10909-2 | CLU | Clusterin |
| P00450 | CP | Ceruloplasmin |
| P02741-1 | CRP | Cysteine-rich protein 1 |
| Q08211 | DHX9 | ATP-dependent RNA helicase A |
| Q02413 | DSG1 | Desmoglein-1 |
| Q16610-4 | ECM1 | Extracellular matrix protein 1 |
| P00748 | F12 | Coagulation factor XII |
| P05160 | F13B | Coagulation factor XIII B chain |
| P00734 | F2 | Prothrombin |
| P23142 | FBLN1 | Fibulin-1 |
| Q9Y6R7 | FCGBP | IgGFc-binding protein |
| O75636-1 | FCN3 | Ficolin-3 |
| P02751-15 | FN1 | Fibronectin |
| P02774-3 | GC | Vitamin D-binding protein |
| P06396 | GSN | Gelsolin |
| P62805 | H4C1 | Histone H4 |
| P69905 | HBA1 | Hemoglobin subunit alpha |
| P68871 | HBB | Hemoglobin subunit beta |
| P02042 | HBD | Hemoglobin subunit delta |
| P00738 | HP | Haptoglobin |
| P00739-2 | HPR | Heparanase |
| P02790 | HPX | Hemopexin |
| P04196 | HRG | Histidine-rich glycoprotein |
| Q86YZ3 | HRNR | Hornerin |
| P35858-2 | IGFALS | Insulin-like growth factor-binding protein complex acid labile subunit |
| P01876 | IGHA1 | Immunoglobulin heavy constant alpha 1 |
| P01859 | IGHG2 | Immunoglobulin heavy constant gamma 2 |
| P01860 | IGHG3 | Immunoglobulin heavy constant gamma 3 |
| P01861 | IGHG4 | Immunoglobulin heavy constant gamma 4 |
| P01871-2 | IGHM | Immunoglobulin heavy constant mu |
| A0A0C4DH31 | IGHV1-18 | Immunoglobulin heavy variable 1-18 |
| P23083 | IGHV1OR15-1 | Immunoglobulin heavy variable 1/OR15-1 |
| A0A0B4J1V0 | IGHV3-15 | Immunoglobulin heavy variable 3-15 |
| P01764 | IGHV3-23 | Immunoglobulin heavy variable 3-23 |
| A0A0B4J1X5 | IGHV3-74 | Immunoglobulin heavy variable 3-74 |
| A0A0B4J1U7 | IGHV6-1 | Immunoglobulin heavy variable 6-1 |
| P01834 | IGKC | Immunoglobulin kappa constant |
| A0A075B6S5 | IGKV1-27 | Immunoglobulin kappa variable 1-27 |
| A0A0C4DH72 | IGKV1-6 | Immunoglobulin kappa variable 1-6 |
| P01594 | IGKV1D-33 | Immunoglobulin kappa variable 1D-33 |
| P06310 | IGKV2-30 | Immunoglobulin kappa variable 2-30 |
| P01614 | IGKV2D-40 | Immunoglobulin kappa variable 2D-40 |
| P01624 | IGKV3-15 | Immunoglobulin kappa variable 3-15 |
| P01619 | IGKV3-20 | Immunoglobulin kappa variable 3-20 |
| A0A0A0MRZ8 | IGKV3D-11 | Immunoglobulin kappa variable 3D-11 |
| A0A0C4DH55 | IGKV3D-7 | Immunoglobulin kappa variable 3D-7 |
| P0DOY2 | IGLC2 | Immunoglobulin lambda constant 2 |
| P0DOY3 | IGLC3 | Immunoglobulin lambda constant 3 |
| P0CF74 | IGLC6 | Immunoglobulin lambda constant 6 |
| A0M8Q6 | IGLC7 | Immunoglobulin lambda constant 7 |
| P15814 | IGLL1 | Immunoglobulin lambda-like polypeptide 1 |
| P01700 | IGLV1-47 | Immunoglobulin lambda variable 1-47 |
| P01701 | IGLV1-51 | Immunoglobulin lambda variable 1-51 |
| P80748 | IGLV3-21 | Immunoglobulin lambda variable 3-21 |
| A0A075B6I9 | IGLV7-46 | Immunoglobulin lambda variable 7-46 |
| A0A075B6I0 | IGLV8-61 | Immunoglobulin lambda variable 8-61 |
| P19827-1 | ITIH1 | Inter-alpha-trypsin inhibitor heavy chain H1 |
| P19823 | ITIH2 | Inter-alpha-trypsin inhibitor heavy chain H2 |
| Q06033-1 | ITIH3 | Inter-alpha-trypsin inhibitor heavy chain H3 |
| Q14624-1 | ITIH4 | Inter-alpha-trypsin inhibitor heavy chain H4 |
| P01591 | JCHAIN | Immunoglobulin J chain |
| P03952 | KLKB1 | Plasma kallikrein |
| P01042 | KNG1 | Kininogen-1 |
| Q5T749 | KPRP | Keratinocyte proline-rich protein |
| P04264 | KRT1 | Keratin, type II cytoskeletal 1 |
| P13645 | KRT10 | Keratin, type I cytoskeletal 10 |
| P02533 | KRT14 | Keratin, type I cytoskeletal 14 |
| P08779 | KRT16 | Keratin, type I cytoskeletal 16 |
| P35908 | KRT2 | Keratin, type II cytoskeletal 2 epidermal |
| P13647 | KRT5 | Keratin, type II cytoskeletal 5 |
| P02538 | KRT6A | Keratin, type II cytoskeletal 6A |
| P04259 | KRT6B | Keratin, type II cytoskeletal 6B |
| P48668 | KRT6C | Keratin, type II cytoskeletal 6C |
| P35527 | KRT9 | Keratin, type I cytoskeletal 9 |
| Q08380 | LGALS3BP | Galectin-3-binding protein |
| P47929 | LGALS7 | Galectin-7 |
| P08519 | LPA | Apolipoprotein(a) |
| O00187-1 | MASP2 | Mannan-binding lectin serine protease 2 |
| P02763 | ORM1 | Alpha-1-acid glycoprotein 1 |
| Q96PD5-2 | PGLYRP2 | N-acetylmuramoyl-L-alanine amidase |
| Q9Y2I7-1 | PIKFYVE | 1-phosphatidylinositol 3-phosphate 5-kinase |
| P00747 | PLG | Plasminogen |
| P27169 | PON1 | Serum paraoxonase/arylesterase 1 |
| A5A3E0 | POTEF | POTE ankyrin domain family member F |
| P02775 | PPBP | Platelet basic protein |
| P07225 | PROS1 | Vitamin K-dependent protein S |
| P07477 | PRSS1 | Trypsin-1 |
| P29074 | PTPN4 | Tyrosine-protein phosphatase non-receptor type 4 |
| P20742 | PZP | Pregnancy zone protein |
| P02753 | RBP4 | Retinol-binding protein 4 |
| P0DJI8 | SAA1 | Serum amyloid A-1 protein |
| P35542 | SAA4 | Serum amyloid A-4 protein |
| P01009-1 | SERPINA1 | Alpha-1-antitrypsin |
| P01011-1 | SERPINA3 | Alpha-1-antichymotrypsin |
| P01008 | SERPINC1 | Antithrombin-III |
| P05546 | SERPIND1 | Heparin cofactor 2 |
| P36955 | SERPINF1 | Pigment epithelium-derived factor |
| P02787 | TF | Serotransferrin, Transferrin |
| P07996 | THBS1 | Thrombospondin-1 |
| P35443 | THBS4 | Thrombospondin-4 |
| P02766 | TTR | Transthyretin |
| P04004 | VTN | Vitronectin |
| P04275 | VWF | von Willebrand factor |
| P0DOX2 | - | Immunoglobulin alpha-2 heavy chain |
| P0DOX4 | - | Immunoglobulin epsilon heavy chain |
| P0DOX5 | - | Immunoglobulin gamma-1 heavy chain |
| P0DOX6 | - | Immunoglobulin mu heavy chain |
| P0DOX7 | - | Immunoglobulin kappa light chain |
| P0DOX8 | - | Immunoglobulin lambda-1 light chain |

**Supplementary table 4. Overlap between the exosomal proteome obtained for ExoQ through mass spectrometry (list of gene names from proteins identified) and the serum-derived exosomal gene list obtained from databases and literature search**. The proteins included were found in at least one individual. Abbreviations: C, Control; AD, Alzheimer’s Disease.

|  |  | **Overlap with exosomal proteome from databases** | | |
| --- | --- | --- | --- | --- |
| **Gene names** | **Protein names** | **C & AD** | **C** | **AD** |
| A1BG | Alpha-1B-glycoprotein | X |  |  |
| A2M | Alpha-2-macroglobulin, Alpha-2-M | X |  |  |
| ACAP2 | Arf-GAP with coiled-coil |  |  | X |
| ACTB | Actin, cytoplasmic 1 |  | X |  |
| ACTBL2 | Beta-actin-like protein 2 |  |  | X |
| AFM | Afamin |  | X |  |
| AGT | Angiotensinogen | X |  |  |
| AHSG | Alpha-2-HS-glycoprotein | X |  |  |
| ALB | Serum albumin | X |  |  |
| AMBP | Protein AMBP | X |  |  |
| ANXA2 | Annexin A2 |  |  | X |
| APCS | Serum amyloid P-component | X |  |  |
| APOA1 | Apolipoprotein A-I | X |  |  |
| APOA2 | Apolipoprotein A-II | X |  |  |
| APOA4 | Apolipoprotein A-IV | X |  |  |
| APOB | Apolipoprotein B receptor | X |  |  |
| APOC3 | Apolipoprotein C-III, Apo-CIII, ApoC-III | X |  |  |
| APOD | Apolipoprotein D | X |  |  |
| APOE | Apolipoprotein E, ApoE | X |  |  |
| APOH | Beta-2-glycoprotein 1 | X |  |  |
| APOL1 | Apolipoprotein L1 | X |  |  |
| APOM | Apolipoprotein M | X |  |  |
| AZGP1 | Zinc-alpha-2-glycoprotein | X |  |  |
| C1QA | Complement C1q subcomponent subunit A | X |  |  |
| C1QB | Complement C1q subcomponent subunit B | X |  |  |
| C1QC | Complement C1q subcomponent subunit C | X |  |  |
| C1R | Complement C1r subcomponent | X |  |  |
| C1S | Complement C1s subcomponent | X |  |  |
| C2 | Complement C2 | X |  |  |
| C3 | Complement C3 | X |  |  |
| C4A | Complement C4-A | X |  |  |
| C4B | Complement C4-B | X |  |  |
| C4BPA | C4b-binding protein alpha chain | X |  |  |
| C4BPB | C4b-binding protein beta chain | X |  |  |
| C5 | Complement C5 | X |  |  |
| C6 | Complement component C6 | X |  |  |
| C7 | Complement component C7 | X |  |  |
| C8A | Complement component C8 alpha chain | X |  |  |
| C8B | Complement component C8 beta chain | X |  |  |
| C8G | Complement component C8 gamma chain | X |  |  |
| C9 | Complement component C9 | X |  |  |
| CD5L | CD5 antigen-like | X |  |  |
| CFB | Complement factor B | X |  |  |
| CFH | Complement factor H | X |  |  |
| CFHR1 | Complement factor H-related protein 1 | X |  |  |
| CFHR2 | Complement factor H-related protein 2 | X |  |  |
| CFI | Complement factor I | X |  |  |
| CFP | Properdin | X |  |  |
| CLEC3B | Tetranectin | X |  |  |
| CLU | Clusterin | X |  |  |
| CNDP1 | Beta-Ala-His dipeptidase |  |  | X |
| CP | Ceruloplasmin | X |  |  |
| CPN2 | Carboxypeptidase N subunit 2 |  | X |  |
| CRP | C-reactive protein |  |  | X |
| DHX9 | ATP-dependent RNA helicase A | X |  |  |
| DSG1 | Desmoglein-1 |  | X |  |
| DST | Dystonin |  | X |  |
| ECM1 | Extracellular matrix protein 1 | X |  |  |
| EFEMP1 | EGF-containing fibulin-like extracellular matrix protein 1 |  | X |  |
| F11 | Coagulation factor XI |  | X |  |
| F12 | Coagulation factor XII | X |  |  |
| F13B | Coagulation factor XIII B chain | X |  |  |
| F2 | Prothrombin | X |  |  |
| FABP5 | Fatty acid-binding protein 5 | X |  |  |
| FBLN1 | Fibulin-1 | X |  |  |
| FCGBP | IgGFc-binding protein |  |  | X |
| FCN3 | Ficolin-3 | X |  |  |
| FN1 | Fibronectin, FN | X |  |  |
| GC | Vitamin D-binding protein, DBP | X |  |  |
| GPX3 | Glutathione peroxidase 3 | X |  |  |
| GSN | Gelsolin | X |  |  |
| HABP2 | Hyaluronan-binding protein 2 | X |  |  |
| HBA1 | Hemoglobin subunit alpha | X |  |  |
| HBB | Hemoglobin subunit beta | X |  |  |
| HBD | Hemoglobin subunit delta |  |  | X |
| HP | Haptoglobin | X |  |  |
| HPR | Glycerate dehydrogenase HPR |  | X |  |
| HPX | Hemopexin | X |  |  |
| HRG | Histidine-rich glycoprotein | X |  |  |
| IGFALS | Insulin-like growth factor-binding protein complex acid labile subunit, ALS | X |  |  |
| IGHA1 | Immunoglobulin heavy constant alpha 1 | X |  |  |
| IGHG2 | Immunoglobulin heavy constant gamma 2 | X |  |  |
| IGHG3 | Immunoglobulin heavy constant gamma 3 | X |  |  |
| IGHG4 | Immunoglobulin heavy constant gamma 4 | X |  |  |
| IGHM | Immunoglobulin heavy constant mu | X |  |  |
| IGHV3-13 | Immunoglobulin heavy variable 3-13 | X |  |  |
| IGHV3-23 | Immunoglobulin heavy variable 3-23 |  | X |  |
| IGHV3-7 | Immunoglobulin heavy variable 3-7 | X |  |  |
| IGHV4-39 | Immunoglobulin heavy variable 4-39 | X |  |  |
| IGKC | Immunoglobulin kappa constant | X |  |  |
| IGKV1-16 | Immunoglobulin kappa variable 1-16 |  | X |  |
| IGKV1-17 | Immunoglobulin kappa variable 1-17 | X |  |  |
| IGKV1-5 | Immunoglobulin kappa variable 1-5 | X |  |  |
| IGKV1D-33 | Immunoglobulin kappa variable 1D-33 | X |  |  |
| IGKV2-30 | Immunoglobulin kappa variable 2-30 | X |  |  |
| IGKV2D-40 | Immunoglobulin kappa variable 2D-40 | X |  |  |
| IGKV3-11 | Immunoglobulin kappa variable 3-11 |  |  | X |
| IGKV3-15 | Immunoglobulin kappa variable 3-15 | X |  |  |
| IGKV3-20 | Immunoglobulin kappa variable 3-20 | X |  |  |
| IGKV4-1 | Immunoglobulin kappa variable 4-1 | X |  |  |
| IGLC2 | Immunoglobulin lambda constant 2 |  |  | X |
| IGLC6 | Immunoglobulin lambda constant 6 |  |  | X |
| IGLC7 | Immunoglobulin lambda constant 7 | X |  |  |
| IGLL1 | Immunoglobulin lambda-like polypeptide 1 |  | X |  |
| IGLV1-40 | Immunoglobulin lambda variable 1-40 | X |  |  |
| IGLV1-44 | Immunoglobulin lambda variable 1-44 |  | X |  |
| IGLV1-47 | Immunoglobulin lambda variable 1-47 | X |  |  |
| IGLV1-51 | Immunoglobulin lambda variable 1-51 | X |  |  |
| IGLV2-11 | Immunoglobulin lambda variable 2-11 |  | X |  |
| IGLV2-14 | Immunoglobulin lambda variable 2-14 |  |  | X |
| IGLV2-23 | Immunoglobulin lambda variable 2-23 | X |  |  |
| IGLV3-19 | Immunoglobulin lambda variable 3-19 |  | X |  |
| IGLV3-21 | Immunoglobulin lambda variable 3-21 | X |  |  |
| IGLV3-25 | Immunoglobulin lambda variable 3-25 | X |  |  |
| ITIH1 | Inter-alpha-trypsin inhibitor heavy chain H1 | X |  |  |
| ITIH2 | Inter-alpha-trypsin inhibitor heavy chain H2 | X |  |  |
| ITIH3 | Inter-alpha-trypsin inhibitor heavy chain H3 | X |  |  |
| ITIH4 | Inter-alpha-trypsin inhibitor heavy chain H4 | X |  |  |
| JCHAIN | Immunoglobulin J chain | X |  |  |
| KLKB1 | Plasma kallikrein | X |  |  |
| KNG1 | Kininogen-1 | X |  |  |
| KRT1 | Keratin, type II cytoskeletal 1 | X |  |  |
| KRT10 | Keratin, type I cytoskeletal 10 | X |  |  |
| KRT14 | Keratin, type I cytoskeletal 14 | X |  |  |
| KRT16 | Keratin, type I cytoskeletal 16 | X |  |  |
| KRT17 | Keratin, type I cytoskeletal 17 |  | X |  |
| KRT2 | Keratin, type II cytoskeletal 2 epidermal | X |  |  |
| KRT24 | Keratin, type I cytoskeletal 24 |  | X |  |
| KRT3 | Keratin, type II cytoskeletal 3 | X |  |  |
| KRT5 | Keratin, type II cytoskeletal 5 | X |  |  |
| KRT6A | Keratin, type II cytoskeletal 6A | X |  |  |
| KRT6B | Keratin, type II cytoskeletal 6B | X |  |  |
| KRT6C | Keratin, type II cytoskeletal 6C | X |  |  |
| KRT85 | Keratin, type II cuticular Hb5 | X |  |  |
| KRT9 | Keratin, type I cytoskeletal 9 | X |  |  |
| LCP2 | Lymphocyte cytosolic protein 2 | X |  |  |
| LGALS3BP | Galectin-3-binding protein |  | X |  |
| LGALS7 | Galectin-7 | X |  |  |
| LPA | Lysophosphatidic acid receptor 1 | X |  |  |
| LRG1 | Rho-GTPase-activating protein LRG1 | X |  |  |
| MASP1 | Mannan-binding lectin serine protease 1 | X |  |  |
| MASP2 | Mannan-binding lectin serine protease 2 |  |  | X |
| MST1 | Hepatocyte growth factor-like protein | X |  |  |
| ORM1 | Protein ORM1 | X |  |  |
| PF4 | Platelet factor 4, PF-4 | X |  |  |
| PGLYRP2 | N-acetylmuramoyl-L-alanine amidase | X |  |  |
| PLEC | Plectin |  | X |  |
| PLG | Plasminogen | X |  |  |
| PON1 | Serum paraoxonase/arylesterase 1 | X |  |  |
| POTEF | POTE ankyrin domain family member F |  |  | X |
| PPBP | Platelet basic protein, PBP | X |  |  |
| PRKDC | DNA-dependent protein kinase catalytic subunit |  | X |  |
| PROS1 | Vitamin K-dependent protein S | X |  |  |
| PRSS1 | Trypsin-1 | X |  |  |
| PZP | Pregnancy zone protein | X |  |  |
| R3HDM2 | R3H domain-containing protein 2 |  |  | X |
| RBP4 | Retinol-binding protein 4 | X |  |  |
| RPS27A | Ubiquitin-40S ribosomal protein S27a |  |  | X |
| S100A8 | Protein S100-A8 |  |  | X |
| SAA1 | Serum amyloid A-1 protein |  |  | X |
| SAA4 | Serum amyloid A-4 protein | X |  |  |
| SERPINA1 | Alpha-1-antitrypsin | X |  |  |
| SERPINA3 | Alpha-1-antichymotrypsin | X |  |  |
| SERPINA5 | Plasma serine protease inhibitor |  | X |  |
| SERPINC1 | Antithrombin-III | X |  |  |
| SERPIND1 | Heparin cofactor 2 | X |  |  |
| SERPINF1 | Pigment epithelium-derived factor, PEDF | X |  |  |
| SERPINF2 | Alpha-2-antiplasmin |  |  | X |
| SERPING1 | Plasma protease C1 inhibitor | X |  |  |
| SFN | 14-3-3 protein sigma |  |  | X |
| TF | Serotransferrin, Transferrin | X |  |  |
| THBS1 | Thrombospondin-1 | X |  |  |
| THBS4 | Thrombospondin-4 |  | X |  |
| TJP2 | Tight junction protein ZO-2 |  |  | X |
| TTN | TTN |  |  | X |
| TTR | Transthyretin | X |  |  |
| VTN | Vitronectin | X |  |  |
| VWF | von Willebrand factor | X |  |  |

**Supplementary table 5. Overlap between the exosomal proteome obtained for ExoS through mass spectrometry (list of gene names from proteins identified) and the serum-derived exosomal gene list obtained from databases and literature search**. The proteins included were found in at least one individual. Abbreviations: C, Control; AD, Alzheimer’s Disease.

|  |  | **Overlap with exosomal proteome from databases** | | |
| --- | --- | --- | --- | --- |
| **Gene names** | **Protein names** | **C & AD** | **C** | **AD** |
| A1BG | Alpha-1B-glycoprotein | X |  |  |
| A2M | Alpha-2-macroglobulin | X |  |  |
| AGT | Angiotensinogen | X |  |  |
| ALB | Serum albumin | X |  |  |
| AMBP | Protein AMBP | X |  |  |
| APCS | Serum amyloid P-component | X |  |  |
| APOA1 | Apolipoprotein A-I | X |  |  |
| APOA4 | Apolipoprotein A-IV | X |  |  |
| APOB | Apolipoprotein B-100 | X |  |  |
| APOD | Apolipoprotein D | X |  |  |
| APOE | Apolipoprotein E | X |  |  |
| APOH | Beta-2-glycoprotein 1 | X |  |  |
| APOL1 | Apolipoprotein L1 | X |  |  |
| APOM | Apolipoprotein M | X |  |  |
| AZGP1 | Zinc-alpha-2-glycoprotein |  | X |  |
| C1QA | Complement C1q subcomponent subunit A | X |  |  |
| C1QC | Complement C1q subcomponent subunit C | X |  |  |
| C1R | Complement receptor type 1 | X |  |  |
| C1S | Complement C1s subcomponent | X |  |  |
| C2 | Complement C2 | X |  |  |
| C3 | Complement C3 | X |  |  |
| C4A | Complement C4-A | X |  |  |
| C4B | Complement C4-B | X |  |  |
| C4BPA | C4b-binding protein alpha chain | X |  |  |
| C4BPB | C4b-binding protein beta chain | X |  |  |
| C5 | Complement C5 | X |  |  |
| C6 | Complement component C6 | X |  |  |
| C7 | Complement component C7 | X |  |  |
| C8B | Complement component C8 beta chain |  |  | X |
| C8G | Complement component C8 gamma chain | X |  |  |
| C9 | Complement component C9 | X |  |  |
| CD5L | CD5 antigen-like | X |  |  |
| CFB | Complement factor B | X |  |  |
| CFH | Complement factor H | X |  |  |
| CFHR1 | Complement factor H-related protein 1 | X |  |  |
| CFHR2 | Complement factor H-related protein 2 |  | X |  |
| CFP | Properdin | X |  |  |
| CLEC3B | Tetranectin | X |  |  |
| CLTC | Clathrin heavy chain 1 |  | X |  |
| CLU | Clusterin | X |  |  |
| CP | Ceruloplasmin | X |  |  |
| CRP | Cysteine-rich protein 1 |  |  | X |
| DHX9 | ATP-dependent RNA helicase A | X |  |  |
| DSG1 | Desmoglein-1 |  |  | X |
| ECM1 | Extracellular matrix protein 1 |  | X |  |
| F12 | Coagulation factor XII | X |  |  |
| F13B | Coagulation factor XIII B chain | X |  |  |
| F2 | Prothrombin | X |  |  |
| FBLN1 | Fibulin-1 | X |  |  |
| FCGBP | IgGFc-binding protein |  |  | X |
| FCN3 | Ficolin-3 |  | X |  |
| FN1 | Fibronectin | X |  |  |
| GC | Vitamin D-binding protein | X |  |  |
| GSN | Gelsolin | X |  |  |
| HBA1 | Hemoglobin subunit alpha | X |  |  |
| HBB | Hemoglobin subunit beta |  |  | X |
| HBD | Hemoglobin subunit delta | X |  |  |
| HP | Haptoglobin | X |  |  |
| HPR | Heparanase |  | X |  |
| HPX | Hemopexin |  |  | X |
| HRG | Histidine-rich glycoprotein | X |  |  |
| HSPB1 | Heat shock protein beta-1 |  |  | X |
| IGFALS | Insulin-like growth factor-binding protein complex acid labile subunit | X |  |  |
| IGHA1 | Immunoglobulin heavy constant alpha 1 | X |  |  |
| IGHG1 | Immunoglobulin heavy constant gamma 1 |  |  | X |
| IGHG2 | Immunoglobulin heavy constant gamma 2 | X |  |  |
| IGHG3 | Immunoglobulin heavy constant gamma 3 | X |  |  |
| IGHG4 | Immunoglobulin heavy constant gamma 4 | X |  |  |
| IGHV3-23 | Immunoglobulin heavy variable 3-23 | X |  |  |
| IGKC | Immunoglobulin kappa constant | X |  |  |
| IGKV1D-33 | Immunoglobulin kappa variable 1D-33 | X |  |  |
| IGKV2-30 | Immunoglobulin kappa variable 2-30 |  | X |  |
| IGKV2D-40 | Immunoglobulin kappa variable 2D-40 | X |  |  |
| IGKV3-15 | Immunoglobulin kappa variable 3-15 | X |  |  |
| IGKV3-20 | Immunoglobulin kappa variable 3-20 | X |  |  |
| IGLC2 | mmunoglobulin lambda constant 2 |  |  | X |
| IGLC6 | Immunoglobulin lambda constant 6 |  |  | X |
| IGLC7 | Immunoglobulin lambda constant 7 |  | X |  |
| IGLL1 | Immunoglobulin lambda-like polypeptide 1 |  | X |  |
| IGLV1-47 | Immunoglobulin lambda variable 1-47 | X |  |  |
| IGLV1-51 | Immunoglobulin lambda variable 1-51 |  | X |  |
| IGLV3-21 | Immunoglobulin lambda variable 3-21 | X |  |  |
| ITIH1 | Inter-alpha-trypsin inhibitor heavy chain H1 | X |  |  |
| ITIH2 | Inter-alpha-trypsin inhibitor heavy chain H2 | X |  |  |
| ITIH3 | Inter-alpha-trypsin inhibitor heavy chain H3 | X |  |  |
| ITIH4 | Inter-alpha-trypsin inhibitor heavy chain H4 | X |  |  |
| JCHAIN | Immunoglobulin J chain | X |  |  |
| KLKB1 | Plasma kallikrein | X |  |  |
| KNG1 | Kininogen-1 | X |  |  |
| KRT1 | Keratin, type II cytoskeletal 1 | X |  |  |
| KRT10 | Keratin, type I cytoskeletal 10 | X |  |  |
| KRT14 | Keratin, type I cytoskeletal 14 | X |  |  |
| KRT16 | Keratin, type I cytoskeletal 16 | X |  |  |
| KRT2 | Keratin, type II cytoskeletal 2 epidermal | X |  |  |
| KRT5 | Keratin, type II cytoskeletal 5 | X |  |  |
| KRT6A | Keratin, type II cytoskeletal 6A | X |  |  |
| KRT6B | Keratin, type II cytoskeletal 6B | X |  |  |
| KRT6C | Keratin, type II cytoskeletal 6C |  | X |  |
| KRT9 | Keratin, type I cytoskeletal 9 | X |  |  |
| LGALS3BP | Galectin-3-binding protein |  | X |  |
| LGALS7 | Galectin-7 |  |  | X |
| LPA | Apolipoprotein(a) | X |  |  |
| MASP2 | Mannan-binding lectin serine protease 2 | X |  |  |
| ORM1 | Alpha-1-acid glycoprotein 1 |  | X |  |
| PGLYRP2 | N-acetylmuramoyl-L-alanine amidase | X |  |  |
| PLG | Plasminogen | X |  |  |
| PON1 | Serum paraoxonase/arylesterase 1 | X |  |  |
| POTEF | POTE ankyrin domain family member F |  |  | X |
| PPBP | Platelet basic protein |  | X |  |
| PRKCH | Protein kinase C eta type |  |  | X |
| PROS1 | Vitamin K-dependent protein S | X |  |  |
| PRSS1 | Trypsin-1 | X |  |  |
| PZP | Pregnancy zone protein | X |  |  |
| RBP4 | Retinol-binding protein 4 | X |  |  |
| SAA1 | Serum amyloid A-1 protein |  |  | X |
| SAA4 | Serum amyloid A-4 protein |  |  | X |
| SERPINA1 | Alpha-1-antitrypsin | X |  |  |
| SERPINA3 | Alpha-1-antichymotrypsin | X |  |  |
| SERPINC1 | Antithrombin-III | X |  |  |
| SERPIND1 | Heparin cofactor 2 |  |  | X |
| SERPINF1 | Pigment epithelium-derived factor |  | X |  |
| SHBG | Sex hormone-binding globulin |  | X |  |
| TF | Serotransferrin, Transferrin | X |  |  |
| THBS1 | Thrombospondin-1 | X |  |  |
| THBS4 | Thrombospondin-4 |  | X |  |
| TTR | Transthyretin | X |  |  |
| VTN | Vitronectin | X |  |  |
| VWF | von Willebrand factor | X |  |  |
| WASL | Neural Wiskott-Aldrich syndrome protein |  |  | X |
